# Smooth and Discrete Cone-Nets

**DOI:** 10.1007/s00025-023-01884-9

**Published:** 2023-03-28

**Authors:** Martin Kilian, Christian Müller, Jonas Tervooren

**Affiliations:** Institute for Discrete Mathematics and Geometry, TU Wien, Wiedner Hauptstraße 8-10/104, 1040 Vienna, Austria

**Keywords:** Cone-nets, conjugate nets, combescure transformation, discrete nets, 53A70, 53A05

## Abstract

Cone-nets are conjugate nets on a surface such that along each individual curve of one family of parameter curves there is a cone in tangential contact with the surface. The corresponding conjugate curve network is projectively invariant and is characterized by the existence of particular transformations. We study properties of that transformation theory and illustrate how several known surface classes appear within our framework. We present cone-nets in the classical smooth setting of differential geometry as well as in the context of a consistent discretization with counterparts to all relevant statements and notions of the smooth setting. We direct special emphasis towards smooth and discrete tractrix surfaces which are characterized as principal cone-nets with constant geodesic curvature along one family of parameter curves.

## Introduction and Preliminaries

### Introduction

Consider a surface and an arbitrary curve on that surface which is not an asymptotic curve. The set of all tangent planes along that surface envelops a generic developable surface. The focus of our paper lies in the investigation of surface parametrizations such that the enveloping developables of one family of parameter curves are *not* generic developable surfaces but merely *cones*. Therefore we use the name *cone-nets* for our parametrizations. Note that our cone-nets are not to be confused with the so called *conical nets* which are discrete nets with planar faces such that at each vertex the adjacent faces around that vertex are in tangential contact with a cone of revolution [1].

The study of our cone-nets has been motivated by the fabrication idea of cladding surfaces with developable strips. The aspects of cladding surfaces with general developable surfaces—not necessarily just cones—have also been investigated in the context of approximating surfaces, for example in [2, 3] in a non-parametrized way, or in the theory of curved crease paper folding (see, e.g., [4]).

Moreover, strip-models of surfaces have been investigated in different contexts. For example strip-models have been used to develop a better understanding of classical smooth differential geometry by replacing smooth surfaces with discrete surfaces. R. Sauer has systematically started to study these discrete counterparts to gain a better understanding of the classical smooth setting [5–7]. By introducing this methodology he laid the ground for the now highly active research field of discrete differential geometry (see, e.g., [8]) to which the second part of the present paper can be counted. In particular R. Sauer’s investigation of surfaces of revolution [5, 6] which he approximated by a collection of cones of revolution can be seen as a first example of a much bigger class of surfaces which are now called semi-discrete surfaces. These surfaces have a smooth and a discrete coordinate direction and are therefore particularly interesting in the context of cladding surfaces from developable strips with the view towards applications [1] but have also led to a new concept in differential geometry [9]. The results of the present paper could be transferred to the semi-discrete setting, but we will refrain from that as our focus lies on the smooth and the purely discrete setting.

Cone-nets form a particular subclass of surface parametrizations namely so called conjugate nets. Conjugate nets have extensively been studied in the nineteenth and twentieth century [10]. Within that class of nets the Kœnigs nets (see, e.g., [8]) form a subclass which are characterized by the existence of transformations. We develop a similar characterization of our cone-nets by the existence of so called *conical Combescure transformations*.

Our cone-nets include and generalize some known surface parametrizations. For example cladding surfaces with strips of just *cylinders* (instead of *cones* in our case) has been studied in [11, 12]. They are considering foliations of surfaces with “planar geodesics” whereas our cone-nets parametrize “spherical curves of constant geodesic curvature”. From the application point of view the special case of cylinders plays an important role. For example, there are industrial glass tempering and bending machines for manufacturing hot and even cold bent glass in the shapes of cylinders. The even more special case of approximating surfaces with just right circular cylinders has been mentioned in [13]. Another example are the discrete canal surfaces in [14] which appear as a special case of our definition of discrete canal surfaces. One further example are the multi-Q-nets [15] which appear in our context as so called double cone-nets. The projective dual of our cone-nets, i.e., networks on surfaces with planar cuves have been studied with a view towards applications in [16]. More investigation in practical methods for the (computational) design of cone-nets will be addressed in a forthcoming paper.

Our paper consists of two corresponding sections where we develop the smooth theory in Sect. 2 which we then discretize in Sect. 3 by following discretization principles as understood in [8].

### Preliminaries

An important role in our paper play developable surfaces. A surface is called *developable* if it is (locally) isometric to the plane, or equivalently, if the Gauss curvature vanishes identically (see e.g., [17]). Developable surfaces devide into three categories: *cylinders*, *cones*, and *tangent surface*.

Developable surfaces are ruled surfaces and can therefore be parameterized in the form

$$\begin{aligned} d(s, t) = c(t) + s e(t), \end{aligned}$$

where $c: \mathbb {R}\supset I \rightarrow \mathbb {R}^3$ is called *directrix* and $e: \mathbb {R}\supset I \rightarrow \mathbb {R}^3\setminus \{0\}$ is the *ruling direction*.

The rulings of a *tangent surface* are the tangent lines of a space curve. This curve is called the *curve of regression* and consists of the singular points of the tangent surface.

The tangent planes along each ruling of a developable surface are identical. In other words, the direction of the normals of *d* do not depend on *s*. Consequently, $d_s \times d_t = e \times (c_t + s e_t)$ points in the same direction independently of *s* if and only if

1 $$\begin{aligned} \det (e, e_t, c_t) = 0. \end{aligned}$$

We say that the developable surface is enveloped by its tangent planes. And also vice versa any generic (and smooth enough) one-parameter family of planes envelopes a developable surface [17].

The *envelope of the tangent planes* along a curve on a surface is the developable surface which is enveloped by the tangent planes along that curve. That envelope degenerates if the curve is an asymptotic curve on the surface.

The focus of our investigation lies primarily in local properties of sufficiently smooth curves and surfaces. We can always assume our surfaces to be parameterized by $f: \mathbb {R}^2 \supset U \rightarrow \mathbb {R}^3$.

We will be working a lot with so called conjugate nets which form a particular class of nets in projective differential geometry but can easily be described in terms of classical differential geometry.

#### Definition 1

A parameterization $f: \mathbb {R}^2 \supset U \rightarrow \mathbb {R}^3$ is called *conjugate* or *a conjugate net* if in each point the mixed partial derivative is parallel to the tangent plane (or vanishes), i.e., there exist $a, b: U \rightarrow \mathbb {R}$ such that $f_{uv} = a f_u + b f_v$.

Conjugate nets are also characterized by the following well known lemma where we consider the envelope of the tangent planes along the *u*-parameter curves (i.e., isolines with fixed parameter *v*) of a smooth net *f*(*u*, *v*).

#### Lemma 1

**Fig. 1:**
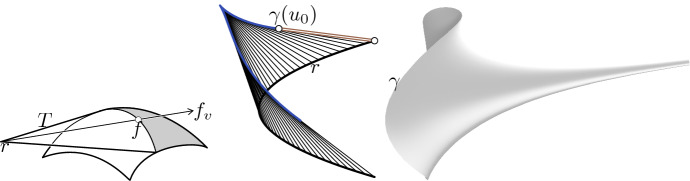
*Left:* A surface *f* and a tangential cone *T* with cone tip *r*. We can interpret the point *r* as a point light and *T* as the light cone consisting of rays connecting the point light with the silhouette. In each point of the silhouette its tangent line and the corresponding ruling of *T* are conjugate tangents. *Center:* A parabola *r* is the base curve of a general tractrix with initial point $\gamma (u_0)$. If $\gamma$ describes a curve, we obtain a generalized tractrix surface, see Example 5 and Sect. 2.5.1. *Right:* A tractrix surface generated by dragging a spherical curve $\gamma$ along a space curve

For any fixed *v* the ruled surface parameterized by (see Fig. 1 left)

$$\begin{aligned} (s, u) \mapsto T(s, u, v):= f(u, v) + s f_v(u, v) \end{aligned}$$

is developable if and only if *f* is a conjugate net.

#### Proof

Developability of *T* is equivalent to the vanishing determinant (cf. Eq. (1))

$$\begin{aligned} \det (f_v, f_{uv}, f_u) = 0, \end{aligned}$$

which is equivalent to *f* being conjugate. $\square$

We will denote the one-parameter family of envelopes of tangent planes by *T*(*s*, *u*, *v*).

## Smooth Cone-Nets

In this section we will define cone-nets which constitute a class of nets, i.e., surface parameterizations, with a one-parameter family of cones in tangential contact with the curves of one family of parameter curves. This class of nets is very rich and exists on every surface at least locally. We will give examples, develop a transformation theory for such nets and classify special cases.

### Smooth Cone-Nets

We start with the definition of cone-nets and illustrate how they are characterized among conjugate nets.

#### Definition 2

We call a net, i.e., a parameterization of a surface, *cone-net* if all envelopes of tangent planes along all *u*-parameter curves (or all *v*-parameter curves) are cones or cylinders. The net is called a *proper cone-net* if all envelopes of tangent planes along all *u*-parameter curves (or all *v*-parameter curves) are cones with a proper cone tip.

If not stated otherwise, we will always assume that the tangential cones are in contact with the surface along the *u*-parameter curves of cone-nets.

In projective geometry cones and cylinders are indistinguishable and since projective transformations keep tangential contact between surfaces we obtain that cone-nets are invariant under projective transformations.

#### Lemma 2

A conjugate net with $f_{uv} = a f_u + b f_v$ is a cone-net, with tangential *cones* along *u*-parameter curves, if and only if $a b = a_u$ and a cone-net, with tangential *cylinders* along *u*-parameter curves, if and only if $a = 0$.

#### Proof

We start with the cylinders. A cylinder is tangent to a *u*-parameter curve of a conjugate net if and only if the partial derivatives $f_v$ are parallel along that curve. This is the case if and only if

$$\begin{aligned} 0 = \partial _u \frac{f_v}{\Vert f_v\Vert } = \frac{\Vert f_v\Vert f_{uv} - f_v \partial _u \Vert f_v\Vert }{\Vert f_v\Vert ^2}, \end{aligned}$$

which is equivalent to $f_{uv} = b f_v$, i.e., if and only if $a = 0$.

Let us now assume that the envelope of tangent planes is not a cylinder, i.e., $a \ne 0$. The curve of regression *r* consists of the singular points of that envelope *T*. It is characterized by those parameters *s* where $T_u \times T_s = 0$. Hence, from

$$\begin{aligned} 0 = T_u \times T_s = (f_u + s f_{uv}) \times f_v = f_u \times f_v + s a f_u \times f_v \end{aligned}$$

we get $s = -\frac{1}{a}$ and consequently $r(u, v) = T(-\frac{1}{a(u, v)}, u, v) = f(u, v) - \frac{1}{a(u, v)} f_v(u, v)$ as curve of regression for each fixed *v*.

However, the envelope of the tangent planes for any fixed *v* is not just an arbitrary tangent surface. It is a cone which means that the curve of regression degenerates to a point—it does not depend on *u*. Therefore, it is a cone if $r_u = 0$, hence,

$$\begin{aligned} 0 = r_u = \Big (\frac{a_u}{a^2} - \frac{b}{a}\Big ) f_v \end{aligned}$$

and consequently $$a b = a_u$$. $$\square $$

From this proof we conclude that the curve of tips of the enveloping cones is parameterized by

2 $$\begin{aligned} r(v):= f - \frac{f_v}{a}. \end{aligned}$$

Any surface can be parameterized, at least locally, by a cone-net. A possible generation of such a net can be explained with a simple geometric construction which, for a special case, was already known to Böklen [18]. Consider a point light in space (the point can also be a point at infinity) which sheds light onto the surface (cf. Fig. 1 left). The light cone consisting of rays connecting the point light with the silhouette is a tangential cone in our sense. Moving the point light along a curve yields a one-parameter family of silhouettes which form *u*-parameter curves of a cone-net. Böklen [18, 10. on p. 69] describes the construction of that special case where the curve of point lights is a straight line.

### Examples of Cone-Nets

There are some well known and commonly used surface parameterizations which are also cone-nets.

#### Example 1

The typical parameterizations of *surfaces of revolution* with meridian curves and parallel circles are cone-nets in both directions. The tangential cones along the parallel circles are rotationally symmetric and have their cone tips on the axis of revolution. The tangential cones along all meridian curves are cylinders.

#### Example 2

*Canal surfaces* are surfaces enveloped by a one-parameter family of spheres. These spheres are in tangential contact with the canal surface along a circle which constitute one family of curvature lines. Along these circles we have cones of revolution in tangential contact with the canal surface along these circles. Therefore, curvature line parameterizations (or principal nets) of canal surfaces are cone-nets. Surfaces of revolution, Dupin cyclides, and tubular surfaces (canal surface with constant radius spheres) are special canal surfaces. We will revisit canal surfaces in the context of principal nets in Sect. 2.5.2.

#### Example 3

*Translational surfaces* *f* are generated as the sum of two curves *g* and *h*:

$$\begin{aligned} f(u, v):= g(u) + h(v). \end{aligned}$$

There are cylinders in tangential contact along both parameter curves. Translational surfaces are therefore cone-nets. Any projective transformation of a translational surface generates a double cone-net (see also Sect. 2.4) whose curves of cone tips lie in a plane which is the image of the plane at infinity.

#### Example 4

Any suitable smooth enough family of planes intersects a quadric in a smooth family of conics. The envelope of tangent planes along such a conic is a cone whose vertex is the projective pole of the plane with respect to the quadric. These poles form the curve *r* of cone tips. The directions of the rulings of the cones are conjugate to the tangents of the conics.

#### Example 5

A tractrix curve is the locus of the endpoint of a stick dragging behind while the other end moves along a straight line. A generalization of that idea is moving one end along an arbitrary space curve *r* instead of a straight line, see Fig. 1 (center). With this method we can obtain a - what we call - *generalized tractrix surface* if we drag each point of a space curve $\gamma$ (Fig. 1 right) along another space curve *r*. In this way each parameter line is a generalized tractrix curve. We will revisit (generalized) tractrix surfaces in Sect. 2.5.1.

As a special case the curve $\gamma$ can lie in its initial position on a sphere with center on the base curve *r*. We refer to them as *tractrix surfaces* since all “sticks” are of the same length. In that case the foliation of parameter curves that is traced out by draging $\gamma$ consists of curves with constant geodesic curvature. The net of parameter curves on a tractrix surface generated this way generalizes the nets from [11, 12]. They are considering foliations of surfaces with planar geodesics. So the analogy is “planar geodesics” vs. “spherical curves of constant geodesic curvature”.

#### Example 6

**Fig. 2:**
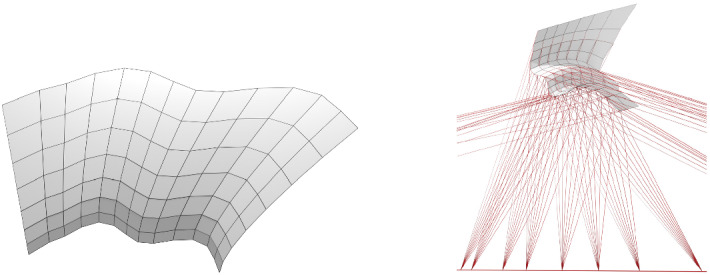
*Left:* A discrete T-surface. All faces are trapezoids. The horizontal edges of each vertical strip in the image are parallel to each other and must therefore lie on a cylinder. *Right:* A projective transformation of the T-surface on the left. The tangential cylinders of the T-surface have been mapped to tangential cones which have their cone tips on a straight line

*T-surfaces* are conjugate nets with planar coordinate curves such that the two families of planes that carry the curves intersect each other orthogonally (see, e.g., [7, 19, 20]). Up to a Euclidean motion every T-surface *f* can be constructed by a one-parameter family of two-dimensional affine transformations $\alpha ^v$ and a curve $c(u) = (x(u), 0, z(u))$ in the *xz*-plane such that

$$\begin{aligned} f(u, v) = \bigg ({ \alpha ^v\big ({x(u)\atop 0}\big ) \atop z(u) }\bigg ), \end{aligned}$$

i.e., every vertical profile curve of *f* is constructed from *c* by a non-uniform scaling (just in *x*-direction) and a rotation around a *z*-parallel axis. See Fig. 2 (left) for a discrete T-surface.

Special sub-classes of T-surfaces are surfaces of revolution and translational surfaces. The tangent planes along a vertical profile curve of a T-surface envelope a cylinder with horizontal rulings. Projective transformations of T-surfaces are cone-nets with all cone tips on a straight line which is the image of the line at infinity (see Fig. 2 right).

### Transformation of Cone-Nets

A classical topic in differential geometry is the transformation of surfaces [8, 10]. A particular focus lies on the transformation of conjugate nets. In this section we introduce a transformation for cone-nets. Thereby, a cone-net is transformed to a parallel cone-net in the following sense.

#### Definition 3

Two nets $f, f^*: \mathbb {R}^2 \supset U \rightarrow \mathbb {R}^3$ are said to be *parallel* or *related by a Combescure transformation*, if at each point corresponding partial derivative vectors are parallel, i.e., $f_u \parallel f_u^*$ and $f_v \parallel f_v^*$. The net $f^*$ is called *Combescure transform* of *f* and vice versa.

#### Theorem 3

Let $f: U \rightarrow \mathbb {R}^3$ be a conjugate net with $f_{uv} = a f_u + b f_v$, for some $a, b: U \rightarrow \mathbb {R}$, where $a \ne 0$. Furthermore, let $\lambda : U \rightarrow \mathbb {R}$ be a function only depending on *v*, i.e., $\lambda _u = 0$. Then for all such $\lambda$ there exists a Combesure transform $f^*$ with

$$\begin{aligned} f_u^* = \lambda f_u \quad \text {and}\quad f_v^* = \Big (\lambda + \frac{\lambda _v}{a}\Big ) f_v \end{aligned}$$

if and only if *f* is a proper cone-net. Furthermore, $f^*$ is a proper cone-net as well and has the following form up to translation

$$\begin{aligned} f^* = \lambda f - \int \lambda _v\Big (f-\frac{f_v}{a}\Big )\, dv = \lambda f - \int \lambda _v r\, dv. \end{aligned}$$

#### Proof

The net $f^*$ exists if and only if the integrability condition $(f_u^*)_v = (f_v^*)_u$ holds. We havebecause $\lambda _u = \lambda _{uv} = 0$. Since the last equation must hold for any $\lambda$, it is equivalent to $a b = a_u$. Consequently, the existence of $f^*$ is equivalent to *f* being a cone-net.

After setting $a^*:= \frac{\lambda _v + a \lambda }{\lambda }$ and $b^*:= \frac{\lambda a_u}{\lambda _v + a \lambda }$ we obtain $f_{uv}^* = a^* f_u^* + b^* f_v^*$. To show that $f^*$ is also a cone-net we must verify that $b^* = \frac{a_u^*}{a^*}$. We have

$$\begin{aligned} \frac{a_u^*}{a^*} = \frac{[(\lambda _{uv} + a_u \lambda + a \lambda _u) \lambda - (\lambda _v + a \lambda ) \lambda _u] \lambda }{\lambda ^2 (\lambda _v + a \lambda )} = \frac{\lambda a_u}{\lambda _v + a \lambda } = b^*. \end{aligned}$$

To obtain the expression for $f^*$ we have to integrate $f_u^* = \lambda f_u$ and $f_v^* = \big (\lambda + \frac{\lambda _v}{a}\big ) f_v$. Since $\lambda$ does not depend on *u*, integration of $f^*_u$ by *u* yields

$$\begin{aligned} f^* = \lambda f + c(v) \end{aligned}$$

with some function *c*(*v*). Differentiating this equation by *v* yields

$$\begin{aligned} f^*_v = \lambda _v f + \lambda f_v + c_v. \end{aligned}$$

Comparing this with the definition of $f_v^*$ implies

$$\begin{aligned} c(v) = - \int \lambda _v\Big (f-\frac{f_v}{a}\Big )\, dv {=} - \int \lambda _v r\, dv, \end{aligned}$$

which is indeed independent of *u* and yields the integral representation of $f^*$.    $\square$

#### Definition 4

We call the Combescure transformations from Theorem 3 which map cone-nets to cone-nets *conical Combescure transformations* or *CCT* for short. For a given cone-net *f* and a non-zero function $\lambda$ we denote the conical Combescure transform by $\mathop {\mathcal {C}}\nolimits _\lambda (f)$.

#### Lemma 4

The set of transformations $\{\mathop {\mathcal {C}}\nolimits _\lambda \mid \lambda \ \text {non-zero function}\}$ is a commutative group with respect to composition. The inverse of $\mathop {\mathcal {C}}\nolimits _{\lambda }$ is given by $\mathop {\mathcal {C}}\nolimits _{\frac{1}{\lambda }}$ and the neutral element is the identity map $\mathop {\mathcal {C}}\nolimits _1$.

#### Proof

The group operation is the composition of maps, hence, we have to show

$$\begin{aligned} \mathop {\mathcal {C}}\nolimits _\mu (\mathop {\mathcal {C}}\nolimits _{\lambda }(f)) = \mathop {\mathcal {C}}\nolimits _{\lambda \mu }(f) \end{aligned}$$

for all non-zero functions $\lambda , \mu$. Since a Combescure transformation determines the transformed surface only up to a translation, we consider the derivatives:

$$\begin{aligned} \mathop {\mathcal {C}}\nolimits _\mu (\mathop {\mathcal {C}}\nolimits _\lambda (f))_u&= \lambda \mu f_u = \mathop {\mathcal {C}}\nolimits _{\lambda \mu }(f)_u \\ \mathop {\mathcal {C}}\nolimits _\mu (\mathop {\mathcal {C}}\nolimits _\lambda (f))_v&= \Big (\mu + \frac{\mu _v}{a^*}\Big ) f_v^* = \Big (\mu + \frac{\mu _v}{\frac{\lambda _v + a \lambda }{\lambda }}\Big ) \Big (\lambda + \frac{\lambda _v}{a}\Big ) f_v \\&= \Big (\lambda \mu + \frac{(\lambda \mu )_v}{a}\Big ) f_v = \mathop {\mathcal {C}}\nolimits _{\lambda \mu }(f)_v, \end{aligned}$$

which is what we wanted to show. $\square$

From Eq. (2) we know that the tips of the enveloping cones lie on the curve $r(v) = f - \frac{f_v}{a}$. The curve of the tips of a *CCT* of *f* can be computed from *r* and $\lambda$.

#### Lemma 5

After a CCT the curve of tips of the one-parameter family of enveloping cones becomes up to translation

3 $$\begin{aligned} r^*(v):= \int \lambda r_v\, dv. \end{aligned}$$

The line segments, connecting the enveloped surface *f* with the curve *r* are scaled by $\lambda$, i.e.,

$$\begin{aligned} r^*(v) - f^*(u, v) = \lambda (v) \cdot (r(v) - f(u, v)). \end{aligned}$$

#### Proof

By the same argument as in the proof of Lemma 2 the curve of the cone tips corresponding to $f^*$ is given by

$$\begin{aligned} r^*(v)&= f^* - \frac{f^*_v}{a^*} = \lambda f - \int \lambda _v\Big (f-\frac{f_v}{a}\Big )\, dv - \frac{\lambda }{\lambda _v + a \lambda } \Big (\lambda + \frac{\lambda _v}{a}\Big ) f_v \\&= \lambda \Big (f-\frac{f_v}{a}\Big ) - \int \lambda _v\Big (f-\frac{f_v}{a}\Big )\, dv = \lambda r - \int \lambda _v r\, dv = \int \lambda r_v\, dv. \end{aligned}$$

For the line segments generating the cones we obtain

$$\begin{aligned} f^* - r^* = \frac{f^*_v}{a^*} = \frac{\lambda }{\lambda _v + a \lambda } \Big (\lambda + \frac{\lambda _v}{a}\Big ) f_v = \lambda \frac{f_v}{a} = \lambda (f - r), \end{aligned}$$

which concludes the proof. $\square$

#### Corollary 6

Conical Combescure transformations have the following properties:

(i) Corresponding *u*-parameter curves of a cone-net *f* and its CCT $f^*$ are related by a homothety.
(ii) For constant $\lambda \in \mathbb {R}\setminus \{0\}$ the corresponding CCT acts on the net as similarity with scaling factor $\lambda$, i.e., $\mathop {\mathcal {C}}\nolimits _\lambda (f) = \lambda f$ up to translation.
(iii) CCTs are preserving angles between parameter curves at corresponding points (since all Combescure tranformations preserve parallelity between tangents in corresponding points).
(iv) If *f* is a principal net (i.e., conjugate and orthogonal), than for any $\lambda$ the corresponding CCT $f^*$ is also a principal net.

Even though cone-nets are projectively invariant, CCTs do not commute with projective transformations. However, affine transformations $\alpha$ do commute with CCTs: 
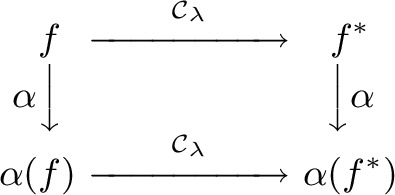


The reason why projective transformations do not commute with CCTs is that in contrast to affine transformations the change of direction of straight lines depends on its location in space. Consider, for example, a projective transformation $\kappa$ that maps a plane $\varepsilon$ to infinity. We can choose *f* with a curve of proper cone tips *r* in $\mathbb {R}^3$ which does not intersect $\varepsilon$, but such that the curve of cone tips $r^*$ of $\mathop {\mathcal {C}}\nolimits _\lambda (f)$ does intersect $\varepsilon$. Then $\kappa \circ f$ only contains proper cones whereas $\kappa \circ \mathop {\mathcal {C}}\nolimits _\lambda (f)$ contains tangential cylinders which we can not get rid of by any CCT.

### Double Cone-Nets

Cone-nets are not symmetric in its definition. The two parameter curves are treated differently. The existence of a tangential cone is assumed only for one family (usually along *u*-parameter curves). However, it poses an interesting question which nets are cone-nets in both directions.

#### Definition 5

We call a net a *double cone-net*, if all envelopes of tangent planes along *both* families of parameter curves are cones or cylinders.

With Lemma 2, a conjugate net $f:U \rightarrow \mathbb {R}^3$ is a double cone-net if and only if the functions *a*, *b* defined by

$$\begin{aligned} f_{uv} = a f_u + b f_v \end{aligned}$$

satisfy

4 $$\begin{aligned} b_v = a_u = ab. \end{aligned}$$

In the following theorem we will show that double cone-nets are so called Kœnigs nets which constitute themselves a special subclass of conjugate nets.

#### Definition 6

A net $f:U \rightarrow \mathbb {R}^3$ is called a *Kœnigs net* if there exists $z: U \rightarrow \mathbb {R}^+$ such that (see, e.g., [8]):

$$\begin{aligned} f_{uv} = (\log z)_v f_u + (\log z)_u f_v. \end{aligned}$$

It is immediately clear from this definition that Kœnigs nets are conjugate nets.

#### Theorem 7

Let $U\subset \mathbb {R}^2$ be a simply connected domain and let $f:U \rightarrow \mathbb {R}^3$ be a conjugate net which is a cone-net w.r.t. the *u*-parameter curves. Then the net *f* is a double cone-net if and only if *f* is a Kœnigs net.

#### Proof

Let us assume *f* is a Kœnigs net and a cone-net w.r.t. the *u*-parameter curves. From the Kœnigs net property we get

$$\begin{aligned} f_{uv} = (\log z)_v f_u + (\log z)_u f_v, \end{aligned}$$

for some $z:U \rightarrow \mathbb {R}^+$. By setting $a:= (\log z)_v$ and $b:= (\log z)_u$ the theorem of Schwarz implies $a_u = b_v$. Since *f* is a cone-net w.r.t. the *u*-parameter curves and by Lemma 2, we further have

$$\begin{aligned} a b = a_u = b_v \end{aligned}$$

which implies that *f* is a double cone-net (cf. Eq. (4)).

Now, let us assume that *f* is a double cone-net. Therefore, *a*, *b* satisfy

$$\begin{aligned} a b = a_u = b_v. \end{aligned}$$

Let us define a vector field $c: U \rightarrow \mathbb {R}^2$ by setting $c:= (b, a)$. On the simply connected domain *U*, the vector field *c* has a potential $p: U \rightarrow \mathbb {R}$ with $\mathop {\text {grad}}p = (p_u, p_v) = c$ if and only if the rotation of *c* is zero. This is the case for double cone-nets since

$$\begin{aligned} \mathop {\text {rot}}(c) = \frac{\partial c_2}{\partial u} - \frac{\partial c_1}{\partial v} = a_u - b_v = ab - ab = 0. \end{aligned}$$

By setting $z:= \exp (p)$ we obtain

$$\begin{aligned} (\log z)_v = p_v = a \\ (\log z)_u = p_u = b \end{aligned}$$

which implies that *f* is a Kœnigs net. $\square$

### Principal Cone-Nets

In this section we will characterize which principal nets are cone-nets and how they can be constructed and what their CCTs are. Furthermore, we will show that the property of a net being a principle cone-net is Möbius invariant.

To this end, let $f:U \rightarrow \mathbb {R}^3$ be a principal net and let $X:= \frac{f_u}{\Vert f_u\Vert }, Y:= \frac{f_v}{\Vert f_v\Vert }, N:= X \times Y$ be a moving frame adapted to *f*. The derivatives of the surface and its frame are given by:

5 $$\begin{aligned} \begin{aligned} f_u&= \alpha X&f_v&= \beta Y \\ X_u&= \kappa Y + c N&X_v&= \eta Y \\ Y_u&= -\kappa X&Y_v&= -\eta X + d N \\ N_u&= -c X&N_v&= - d Y \end{aligned} \end{aligned}$$

for some smooth functions $\alpha , \beta , c,d, \kappa , \eta$. The structure equations of the framed surface read:

6 $$\begin{aligned} \alpha _v&=-\beta \kappa \quad{} & {} \text {structure equation 1} \end{aligned}$$

7 $$\begin{aligned} \beta _u&= \alpha \eta \quad{} & {} \text {structure equation 2} \end{aligned}$$

8 $$\begin{aligned} \kappa _v -\eta _u&= cd \quad{} & {} \text {Gauss equation} \end{aligned}$$

9 $$\begin{aligned} \kappa d&= c_v \quad{} & {} \text {Gauss--Codazzi equation 1} \end{aligned}$$

10 $$\begin{aligned} \eta c&= d_u{} & {} \text {Gauss--Codazzi equation 2} \end{aligned}$$

The symmetry equation is always satisfied for conjugate nets. By the fundamental theorem of parameterized surfaces, any set of functions $\alpha , \beta , \kappa ,\eta ,c,d$, that satisfies the structure equations (6)–(10), determines a principal net and the surface is unique up to rigid motions.

The geodesic curvature of a curve on a surface measures the curvature of the curve projected into the tangent plane. We obtain the geodesic curvature for the *u*-parameter curves by

$$\begin{aligned} k_g^u = \frac{\langle f_u \times f_{uu}, N\rangle }{\Vert f_u\Vert ^3} \overset{(5)}{=} \frac{\kappa }{\alpha } \quad \text {and analogously}\quad k^v_g = \frac{\eta }{\beta }, \end{aligned}$$

for the *v*-parameter curves. The principal curvatures are given by $\kappa _1:= -\frac{c}{\alpha }$ and $\kappa _2:= -\frac{d}{\beta }$.

With respect to the frame, the mixed derivative of the surface *f* is given by

$$\begin{aligned} f_{uv} = \alpha _v X + \alpha \eta Y = -\beta \kappa X + \beta _u Y. \end{aligned}$$

Hence, the functions *a* and *b*, defined by $f_{uv} = a f_u +b f_v$, can be expressed as

11 $$\begin{aligned} a = \frac{\alpha _v}{\alpha } = \frac{-\beta \kappa }{\alpha }, \quad \quad b = \frac{\alpha \eta }{\beta } = \frac{\beta _u}{\beta }. \end{aligned}$$

#### Lemma 8

Let *f* be a principal net. Then the following conditions are equivalent:

(i) The net *f* is a cone-net.
(ii) The geodesic curvature of the *u*-parameter curves (or *v*-parameter curves) is constant for each curve.
(iii) The *u*-parameter curves (or *v*-parameter curves) are spherical or planar and these spheres or planes intersect the surface orthogonally.

#### Proof

(i) $\Rightarrow$ (ii): If the net is a cone-net along the *u*-parameter curves, then Lemma 2 implies $a_u = a b$. Differentiating Eq. (11) yields

$$\begin{aligned} a_u = \Big (\frac{-\beta \kappa }{\alpha }\Big )_u = -\beta _u \frac{\kappa }{\alpha } - \beta \Big (\frac{\kappa }{\alpha }\Big )_u = a b - \beta (k^u_g)_u, \end{aligned}$$

which implies that the geodesic curvature is constant.

(ii) $\Rightarrow$ (iii): If the constant geodesic curvature of a *u*-parameter curve of a principle cone-net is non-zero, then the function

12 $$\begin{aligned} r(v):= f - \frac{f_v}{a} = f + \frac{\alpha }{\kappa } Y = f + \frac{1}{k^u_g} Y, \end{aligned}$$

is independent of *u*, i.e.

$$\begin{aligned} r_u = f_u + \frac{\alpha }{\kappa } Y_u \overset{(5)}{=} \alpha X - \frac{\alpha }{\kappa }\kappa X = 0. \end{aligned}$$

Hence the *u*-curve is contained in a sphere *S*(*v*) (see below). If the geodesic curvature vanishes, i.e., $k_g^u = 0$, then $\kappa = 0$ and Eq. (5) implies $Y_u = 0$. Therefore, the *v*-derivative vectors along any such *u*-parameter curve have the same direction $f_v(u, v) = \beta (u, v) Y(v)$, and define a cylinder. In this case, the *u*-curve is contained in a plane *S*(*v*). Consequently, we set

$$\begin{aligned} S(v):= {\left\{ \begin{array}{ll} \{x \in \mathbb {R}^3 \mid \Vert x - r(v)\Vert ^2 = (k^u_g)^{-2}\} &{}\text {if}\ k_g^u \ne 0, \\ \{x \in \mathbb {R}^3 \mid \langle x - f(u_0, v), Y(v) \rangle = 0\} \quad \text {for some}\ u_0 &{}\text {if}\ k_g^u = 0. \end{array}\right. } \end{aligned}$$

(iii) $\Rightarrow$ (i): If a *u*-parameter curve lies on a sphere which intersects *f* orthogonally, then the tangents of the *v*-parameter curves along this *u*-parameter curve must pass through the center of the sphere (since $f_u \perp f_v$ for principal nets). The argument for *u*-parameter curves being planar works analogously.    $\square$

#### Definition 7

We call the spheres or planes *S*(*v*) *geodesic curvature spheres*.

#### Remark 1

(Cauchy data for principle cone-nets) The *v*-parameter curves of a principle cone-net *f* are orthogonal trajectories of the geodesic curvature spheres *S*(*v*). In particular, any principle cone-net is uniquely determined by its geodesic curvature spheres *S*(*v*) and a spherical curve $\gamma (u) \in S(v_0)$.

#### Lemma 9

The property of a net being a principle cone-net is invariant under Möbius transformations.

#### Proof

Möbius transformations map principle nets to principle nets (see, e.g., [8]). Further, Möbius transformations map spheres and planes to spheres or planes. Lemma 8 implies that principle nets are cone-nets if and only if they have one family of spherical parameter curves whose spheres intersect the surface orthogonally. Consequently, Möbius transformations map principle cone-nets to principle cone-nets. $\square$

#### Tractrix Surfaces

Given a curve $\gamma : \mathbb {R}\supset I \rightarrow \mathbb {R}^3$ and a space curve $r: \mathbb {R}\supset J \rightarrow \mathbb {R}^3$, we can define a surface $f: U \rightarrow \mathbb {R}^3$ with $U = I \times J$ by solving an initial value problem for every fixed *u*:

$$\begin{aligned} f(u, v_0)&= \gamma (u) \quad v_0 \in J, \\ \frac{f_v(u,v)}{\Vert f_v(u,v)\Vert }&= \frac{f(u,v) - r(v)}{\Vert f(u,v) - r(v)\Vert }. \end{aligned}$$

If the curve *r*(*v*) is a straight line, then the solution for any *u* of the above differential equation is the well known *tractrix*. If furthermore, the initial curve is a circle in a plane orthogonal to that straight line, then the surface is the pseudosphere. For arbitrary curves $\gamma$ and *r* we call the resulting surface a *generalized tractrix surface*. If $\gamma$ is a curve on a sphere with its center on the base curve *r*, we call it simply *tractrix surface* since all *v*-parameter lines are tractrices with the same “stick” length. The following lemma is an immediate consequence of the definition.

##### Lemma 10

Every tractrix surface consists of orthogonal trajectories of a one-parameter family of spheres with constant radii.

##### Lemma 11

Let $f: U \rightarrow \mathbb {R}^3$ be a principal cone-net. Then a CCT with parameter $\lambda$ maps *f* to a principal cone-net $f^*$. The geodesic curvatures of the *u*-parameter curves of the related surfaces *f* and $f^*$ satisfy

$$\begin{aligned} \vert k_g^{u\, *}\vert = \frac{\vert k_g^{u}\vert }{\vert \lambda \vert }. \end{aligned}$$

##### Proof

Let $f: U \rightarrow \mathbb {R}^3$ be a principal cone-net and $f^*$ its conical Combescure transform, i.e.,

$$\begin{aligned} f_u^* = \lambda f_u = \lambda \alpha X = \alpha ^* X^* \quad \text {and}\quad f_v^* = \Big (\lambda + \frac{\lambda _v}{a}\Big ) f_v = \Big (\lambda + \frac{\lambda _v}{a}\Big ) \beta Y = \beta ^* Y^*. \end{aligned}$$

Since the tangents of the two nets are parallel at corresponding points, the frame $X^*, Y^*, N^*$ of $f^*$ is parallel to the frame of *f*. Since the functions $\eta , \kappa , c$ and *d* only depend on the frame, they do not change under the CCT up to a possible sign change. Therefore, $f^*$ is a principle net if and only if *f* is a principal net (which we already saw in Corollary 6(iv)) and the absolute value of the geodesic curvature of the *u*-parameter curves is given by

$$\begin{aligned} \vert k_g^{u\, *}\vert = \Big \vert \frac{\kappa ^*}{\alpha ^*}\Big \vert = \Big \vert \frac{\kappa }{\lambda \alpha }\Big \vert = \Big \vert \frac{k_g^{u}}{\lambda }\Big \vert . \end{aligned}$$

which is what we wanted to show. $\square$

##### Corollary 12

A net is a principal cone-net with non-vanishing geodesic curvature if and only if it is a CCT of a tractrix surface.

#### Canal Surfaces

*Canal surfaces* are surfaces enveloped by a one-parameter family of spheres

$$\begin{aligned} \{x \in \mathbb {R}^3 \mid \Vert x-m(v)\Vert ^2 = R^2(v)\}. \end{aligned}$$

These spheres are in tangential contact with the canal surface along circles which constitute one family of curvature lines. These so called *generating circles* are given by the intersection of the above spheres with the planes

$$\begin{aligned} \{x \in \mathbb {R}^3 \mid \langle x - m(v), -m_v(v)\rangle = R_v(v) R(v)\}. \end{aligned}$$

Hence the centers of the circles and their radii are given by

$$\begin{aligned} c(v):= m(v) - \frac{R_v(v) R(v)}{\Vert m_v(v)\Vert ^2} m_v(v), \quad \quad \rho (v):= \frac{R(v)}{\Vert m_v(v)\Vert }\sqrt{\Vert m_v(v)\Vert ^2 - R_v(v)^2}. \end{aligned}$$

**Fig. 3:**
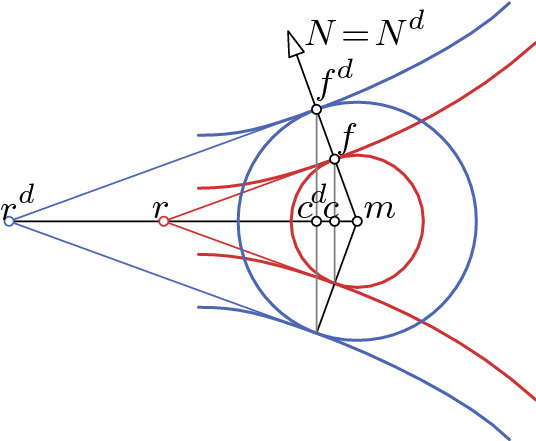
A sphere and its tangential cone of a canal surface *f* and its offset surface $f^d = f + d \cdot N$

Along these circles there are cones of revolution in tangential contact with the surface. The curve of cone tips can be computed by using simple trigonometry (see Fig. 3)

$$\begin{aligned} r(v) = m(v) - \frac{R(v)}{R_v(v)} m_v(v). \end{aligned}$$

The principle curvature of the circular curvature lines is given by $\kappa _1 = -\frac{c}{\alpha } = R(v)^{-1}$. It is well known that a surface is a canal surface if and only if one of the principle curvatures is constant along its curvature line. Surfaces of revolution, Dupin cyclides, and tubular surfaces (canal surface with constant radius spheres) are special canal surfaces.

##### Theorem 13

A principal cone-net $f:U \rightarrow \mathbb {R}^3$ is a canal surface if and only if its Gauss map $N: U \rightarrow \mathbb {S}^2$ is a cone-net.

##### Proof

We compute the mixed derivative of the Gauss map, using the frame equations (5)–(10):

$$\begin{aligned} N_{uv} = -c_v X -c \eta Y = \frac{c_v}{c} N_u + \frac{c \eta }{d} N_v = \frac{\kappa d}{c} N_u + \frac{d_u}{d} N_v, \end{aligned}$$

therefore,

$$\begin{aligned} \quad \quad N_{uv} = {\tilde{a}} N_u + {\tilde{b}} N_v, \quad \text {where}\quad {\tilde{a}} := \frac{\kappa d}{c} = \frac{c_v}{c} \quad \text {and}\ \quad {\tilde{b}} := \frac{c \eta }{d} = \frac{d_u}{d}. \end{aligned}$$

The Gauss map is a cone-net if and only if ${\tilde{a}}_u = {\tilde{a}} {\tilde{b}}$. We have

$$\begin{aligned} {\tilde{a}}_u = \frac{\kappa _u d + \kappa d_u}{c} - \frac{\kappa d c_u}{c^2} = {\tilde{a}}\Big (\frac{\kappa _u}{\kappa } - \frac{c_u}{c}\Big ) + {\tilde{a}}{\tilde{b}}. \end{aligned}$$

Therefore, the Gauss map is a cone-net if and only if $\frac{\kappa _u}{\kappa } = \frac{c_u}{c}$. Since we assumed that *f* is a principle cone-net, the geodesic curvature of the *u*-parameter curves is constant:

$$\begin{aligned} \Big (\frac{\kappa }{\alpha }\Big )_u = 0 \Longleftrightarrow \Big (\frac{\kappa _u}{\kappa } = \frac{\alpha _u}{\alpha } \text { or } \kappa = 0\Big ). \end{aligned}$$

Hence the principle curvature $\kappa _1 = \frac{c}{\alpha }$ is constant along the *u*-parameter curves and *f* is a canal surface if and only if *N* is a cone-net. $\square$

##### Lemma 14

The Gaussian image of any principal net of a canal surface is its CCT for $\lambda = \kappa _1 = -\frac{c}{\alpha }$, which is the principle curvature of the generating circles. Further, any CCT of a canal surface is again a canal surface.

##### Proof

For any principle net the formula of Rodrigues (see, e.g., [17]) implies that the partial derivatives of the Gauss map and the surface are parallel, i.e.,

$$\begin{aligned} N_u = \kappa _1 f_u = -\frac{c}{\alpha } f_u, \quad \quad N_v = \kappa _2 f_v = -\frac{d}{\beta } f_v. \end{aligned}$$

If *f* is a canal surface, one of the principle curvatures is constant along its curvature line. We assume $0 = (\kappa _1)_u = -(\frac{c}{\alpha })_u$. Under the CCT with function $\kappa _1$ the *u*-derivative changes according to

$$\begin{aligned} \mathop {\mathcal {C}}\nolimits _{\kappa _1}(f)_u&= -\frac{c}{\alpha } f_u = \kappa _1 f_u = N_u. \end{aligned}$$

For a principle cone-net we further have $a = \frac{\alpha _v}{\alpha }$ (see Eq. (11)). For the *v*-derivative we obtain

$$\begin{aligned} \mathop {\mathcal {C}}\nolimits _{\kappa _1}(f)_v&= \Big (\kappa _1 + \frac{(\kappa _1)_v}{a} \Big )f_v = \Big (-\frac{c}{\alpha } + \Big (-\frac{c_v}{\alpha } + \frac{\alpha _v c}{\alpha ^2}\Big )\frac{\alpha }{\alpha _v} \Big )f_v \\&= -\frac{c_v}{\alpha _v}f_v \! \overset{(6)(9)}{=} \! -\frac{d}{\beta } f_v = N_v, \end{aligned}$$

and therefore $$\mathop {\mathcal {C}}\nolimits _{\kappa _1}(f) = N$$.

For the second part of the proof note that parallel related surfaces have the same Gauss map. Therefore, the Gauss map of every CCT of a canal surface is a cone-net and the transformed surface is a canal surface. $\square$

The offset surface $f^d:= f + d\,N$, for $d \in \mathbb {R}$ of a canal surface is a canal surface itself where the corresponding spheres have the same centers as for *f* but radii $R(v) + d$ (see Fig. 3). Since any surface and its offset surfaces have the same normals, we can use Lemma 14 to obtain the following corollary.

##### Corollary 15

Let *f* be a canal surface and $\kappa _1(v)$ the principle curvature of the generating circles. The offset $f^d$ is up to translation a CCT of *f* for $\lambda = 1 + d\,\kappa _1$

Darboux proved in [21] that a principle net, whose coordinate curves have constant geodesic curvature, is Möbius equivalent to a surface of revolution, cone or cylinder. With Lemma 8 this implies the following theorem of which an independent proof in our framework can be found in the appendix.

##### Theorem 16

(Darboux [21]) Every double principal cone-net is Möbius equivalent to a surface of revolution, cone or cylinder.

An immediate consequence of Theorem 16 is Vessiot’s Theorem.

##### Theorem 17

(Vessiot [22]) Away from umbilical points every isothermic canal surface is locally Möbius equivalent to a surface of revolution, cone or cylinder.

##### Proof

Away from umbilical points canal surfaces admit a cone-net parameterization. If this net is isothermic, i.e., a principle Kœnigs net, it is a double cone-net and we can apply Theorem 16 which concludes the proof. $\square$

## Discrete Cone-Nets

In this section we discretize our smooth theory of cone-nets. We follow discretization principles as understood in [8]. It turns out that all relevant theorems from Sect. 2 can be discretized.

It is important to point out a possible confusion between notions. So called (discrete) *conical nets* have been introduced and investigated in [1, 8]. However, they refer to a discretization of curvature line parameterizations (which belongs to Laguerre geometry). There, all faces around a vertex are in tangential contact with a cone of revolution. In contrast to that in our case the cones are in tangential contact with the surface along entire parameter curves.

### Discrete Cone-Nets

In this section, we discretize the smooth theory of Sect. 2. The discrete analogue of a parameterized surface is a quadrilateral net

$$\begin{aligned} f : U \subset \mathbb {Z}^2&\longrightarrow \mathbb {R}^3 \\ (i, j)&\longmapsto f_{ij}. \end{aligned}$$

Its discrete derivatives will be described by its edge vectors with difference operators

$$\begin{aligned} \delta _i f_{ij} = f_{i + 1, j} - f_{ij} \quad \text {and}\quad \delta _j f_{ij} = f_{i, j + 1} - f_{ij}, \quad \quad \text {for all}\ (i, j) \in U. \end{aligned}$$

#### Definition 8

A quadrilateral net is called *conjugate* if all quadrilaterals $q_{ij}$ are planar (see, e.g., [8]).

In complete analogy to the smooth case, conjugate nets are characterized by the property that the mixed derivative lies in the span of the partial derivatives (cf. [8]):

#### Lemma 18

A quadrilateral net is conjugate if and only if there exist functions $a, b: U \rightarrow \mathbb {R}$ such that

$$\begin{aligned} \delta _i \delta _j f_{ij} = a_{ij} \delta _i f_{ij} + b_{ij} \delta _j f_{ij}. \end{aligned}$$

**Fig. 4:**
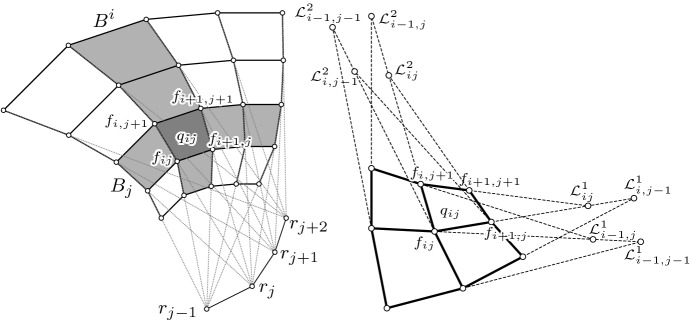
*Left:* A discrete cone-net. *Right:* Laplace points of a conjugate net

Since $\delta _i \delta _j f_{ij} = \delta _i f_{i, j + 1} - \delta _i f_{ij} = \delta _j f_{i + 1, j} - \delta _j f_{ij}$, we immediately obtain

13 $$\begin{aligned} \delta _j f_{i + 1, j}&= a_{ij} \delta _i f_{ij} + (b_{ij} + 1) \delta _j f_{ij} \end{aligned}$$

14 $$\begin{aligned} \delta _i f_{i, j + 1}&= (a_{ij}+1) \delta _i f_{ij} + b_{ij} \delta _j f_{ij} \end{aligned}$$

which we will use later. We will denote the quadrilaterals by

15 $$\begin{aligned} q_{ij}:= (f_{ij}, f_{i + 1, j}, f_{i + 1, j + 1}, f_{i, j + 1}), \end{aligned}$$

and for every *j* we define the *horizontal* strip $B_j:= \{q_{ij} \mid (i, j) \in U\}$ and for every *i* the *vertical* strip $B^i:= \{q_{ij} \mid (i, j) \in U\}$, see Fig. 4 (left). Let $L_{ij}$ denote the straight line that contains the edge $f_{ij} f_{i, j + 1}$, i.e.,

$$\begin{aligned} L_{ij} = f_{ij} + \mathbb {R}\delta _j f_{ij}. \end{aligned}$$

If all lines $L_{ij}$ are concurrent for all *i* and a fixed *j*, they generate a (discrete) cone (i.e., a pyramid) $T_j$. The parameter curves $i \mapsto f_{ij} =: \gamma ^j(i)$ lie on the cones $T_j$ and $T_{j - 1}$. The cone tip of the cone $T_j$ is denoted by $r_j$.

#### Definition 9

A discrete conjugate net $f: U \subset \mathbb {Z}^2 \rightarrow \mathbb {R}^3$ is called *a cone-net* if all horizontal or all vertical strips are contained in a discrete cone or cylinder. The net is called a *proper cone-net* if each horizontal or each vertical strip is contained in a proper cone with a proper cone tip. See Fig. 4 (left) for an illustration.

If not stated otherwise, we will always assume, that the horizontal strips $B_j$ of a discrete cone-net are contained in a cone. The following lemma is a discrete analogue of Lemma 2.

#### Lemma 19

A conjugate net $f: U \subset \mathbb {Z}^2 \rightarrow \mathbb {R}^3$ is a cone-net with discrete *cones* along horizontal strips if and only if the functions $a, b: U \rightarrow \mathbb {R}$ defined in Lemma 18 satisfy

$$\begin{aligned} b_{ij} = \frac{a_{ij} - a_{i - 1, j}}{a_{i - 1, j}} = \frac{a_{ij}}{a_{i - 1, j}} - 1, \end{aligned}$$

and a cone-net with discrete *cylinders* along horizontal strips if and only if $a_{ij} = 0$. If the surface is a cone-net, then the tips of the cones are given by

16 $$\begin{aligned} r_j = f_{ij} - \frac{1}{a_{i - 1, j}} \delta _j f_{ij} = f_{ij} - \frac{b_{ij} + 1}{a_{ij}} \delta _j f_{ij}. \end{aligned}$$

#### Proof

Note that the horizontal strip $B_j$ lies on a cylinder if and only if the edge vectors $\delta _j f_{ij}$ are parallel for all *i*. Equation (13) implies that this is the case if and only if $a_{ij}= 0$ for all *i*.

Suppose now the horizontal strips lie on proper cones. Consider the lines $L_{ij}$. The surface is conjugate if and only if $L_{ij}$ and $L_{i + 1, j}$ intersect each other in a point $r_{ij}$ for all $(i, j) \in U$. We compute this intersection point $r_{ij}$ using Eq. (13)The intersection point $r_{ij}$ is therefore given by:

$$\begin{aligned} r_{ij} = f_{ij} - \frac{b_{ij}+1}{a_{ij}} \delta _j f_{ij} = f_{i + 1, j} - \frac{1}{a_{ij}} \delta _j f_{i + 1, j}. \end{aligned}$$

The surface is a cone-net if $r_{ij}$ is independent of *i*, i.e., $r_{ij} = r_{i + 1, j}$ for all *i*. Consequently, in that case we have

$$\begin{aligned} f_{i + 1, j} - \frac{1}{a_{ij}} \delta _j f_{i + 1, j} = r_{ij} = r_{i + 1, j} = f_{i + 1, j} - \frac{b_{i + 1, j} + 1}{a_{i + 1, j}} \delta _j f_{i + 1, j}, \end{aligned}$$

and therefore

$$\begin{aligned} b_{i + 1, j} = \frac{a_{i + 1, j}}{a_{ij}} - 1 \quad \text {for all\ } i, \end{aligned}$$

which concludes the proof. $\square$

#### Remark 2

For a quadrilateral $q_{ij} = (f_{ij}, f_{i + 1, j}, f_{i + 1, j + 1}, f_{i, j + 1})$ of a conjugate net *f*, the *Laplace points* are defined as the intersection points of opposite edges (see Fig. 4 right), i.e.,

$$\begin{aligned} \mathcal {L}^1_{ij}&:= (f_{ij} \vee f_{i + 1, j}) \cap (f_{i, j + 1} \vee f_{i + 1, j + 1}), \\ \mathcal {L}^2_{ij}&:= (f_{ij} \vee f_{i, j + 1}) \cap (f_{i + 1, j} \vee f_{i + 1, j + 1}). \end{aligned}$$

The Laplace points define (possibly degenerate) conjugate nets themselves which are called the *Laplace transforms of f* (see, e.g., [8]). Since opposite edges might be parallel, these nets are not necessarily contained in $\mathbb {R}^3$ but in the projective space $\mathbb {P}(\mathbb {R}^3)$, i.e., $\mathcal {L}^k: \mathbb {Z}^2 \rightarrow \mathbb {P}(\mathbb {R}^3)$. Note that the net *f* is a cone-net if and only if one Laplace transform degenerates to a polygon.

### Transformation of Discrete Cone-nets

Several aspects of the classical theory on transformations of surfaces have been discretized (see e.g., [23]). Hereby, the transformation of discrete conjugate nets plays a prominent role. We add to that theory the characterization of discrete cone-nets via a discrete transformation theory. Our transformations of discrete nets behave analogously to their smooth counterparts.

#### Definition 10

Two discrete conjugate nets $f, f^*: \mathbb {Z}^2 \supset U \rightarrow \mathbb {R}^3$ are said to be *parallel* or *related by a Combescure transformation*, if for all $(i, j) \in U$ corresponding edge vectors are parallel, i.e., $\delta _i f_{ij} \parallel \delta _i f_{ij}^*$ and $\delta _j f_{ij} \parallel \delta _j f_{ij}^*$. Each of them is called *Combescure transform* of the other. A Combescure transformation is called *a cone-net*, if the transformation of a discrete cone-net is a cone-net.

**Fig. 5:**
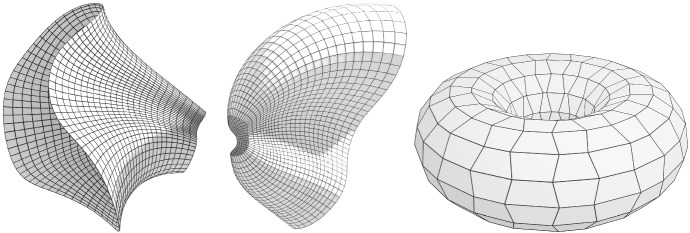
*Left and center:* A pair of discrete conical Combescure transforms. A discrete tractrix surface (*left*) has been transformed with a negative valued function $\lambda$ to a cone-net (*center*) which is not a tractrix surface anymore. Both nets are principal nets. *Right:* A Kœnigs nets with one family of strips being cylinders but which are not double cone-nets

The following theorem about discrete cone-nets preserving Combescure transformations is a discrete analogue of Theorem 3 (see also Fig. 5 left and center).

#### Theorem 20

Let $U = \{0, \ldots , m\} \times \{0, \ldots , n\} \subset \mathbb {Z}^2$ and let $f:U \rightarrow \mathbb {R}^3$ be a discrete conjugate net with $\delta _i\delta _j f_{ij} = a_{ij} \delta _i f_{ij} + b_{ij} \delta _j f_{ij}$ where $a_{ij} \ne 0$. Furthermore, let $\lambda : U \rightarrow \mathbb {R}$ be a function only depending on *j*, i.e., $\delta _i\lambda _{ij} = 0$. We will write $\lambda _j$ instead of $\lambda _{ij}$.

Then *f* is a cone-net if and only if for every such function $\lambda$ there exists a Combescure transform $f^*:U \rightarrow \mathbb {R}^3$ of *f* with:

17 $$\begin{aligned} \delta _i f_{ij}^* = \lambda _j \delta _i f_{ij}, \quad \quad \delta _j f_{ij}^* = \Big (\lambda _{j + 1} + \frac{\lambda _{j + 1} - \lambda _j}{a_{i - 1, j}}\Big ) \delta _j f_{ij}. \end{aligned}$$

If the net *f* is a cone-net, the Combescure transform $f^*$ is a cone-net as well and its vertices are parameterized by

18 $$\begin{aligned} f_{ij}^* = \lambda _j f_{ij} - \sum _{k = 0}^{j - 1} (\lambda _{k + 1} - \lambda _k) r_k, \end{aligned}$$

where $r_j$ are the tips of the cones of the original cone-net *f*.

#### Proof

By Lemma 19 the net *f* is a cone-net if and only if $b_{ij} = \frac{a_{ij}}{a_{i - 1, j}} - 1$ for all $(i, j) \in U$. The set of edge vectors $\delta _i f_{ij}^*$, $\delta _j f_{ij}^*$ can be integrated to a net $f^*: U \rightarrow \mathbb {R}^3$ if and only if the boundary edges of every quadrilateral sum up to zero. Using Eqs. (13) and (14) we compute the sum of the boundary edges of the quadrilateral $q_{ij}^*$:

$$\begin{aligned}&\delta _i f_{ij}^* + \delta _j f_{i + 1, j}^* - \delta _i f_{i, j + 1}^* - \delta _j f_{ij}^* \\&\quad = \lambda _j \delta _i f_{ij} + \Big (\lambda _{j + 1} + \frac{\lambda _{j + 1} - \lambda _j}{a_{ij}}\Big )\delta _j f_{i + 1, j} \\&\qquad -\, \lambda _{j + 1} \delta _i f_{i, j + 1} - \Big (\lambda _{j + 1} + \frac{\lambda _{j + 1} - \lambda _j}{a_{i - 1, j}}\Big ) \delta _j f_{ij} \\&\quad = \Big (\lambda _j + \Big (\lambda _{j + 1} + \frac{\lambda _{j + 1} - \lambda _j}{a_{ij}}\Big ) a_{ij} - \lambda _{j + 1}(a_{ij} + 1)\Big ) \delta _i f_{ij} \\&\qquad +\, \Big (\Big (\lambda _{j + 1} + \frac{\lambda _{j + 1} - \lambda _j}{a_{ij}}\Big ) (b_{ij} + 1) - \lambda _{j + 1} b_{ij} - \Big (\lambda _{j + 1} + \frac{\lambda _{j + 1} - \lambda _j}{a_{i - 1, j}}\Big )\Big ) \delta _j f_{ij} \\&\quad = (\lambda _{j + 1} - \lambda _j) \Big (\frac{b_{ij} + 1}{a_{ij}} - \frac{1}{a_{i - 1, j}}\Big ) \delta _j f_{ij}. \end{aligned}$$

The edge cycle is closed and $f^*$ well defined for every choice of $\lambda$ if and only if $b_{ij} = \frac{a_{ij}}{a_{i - 1, j}} - 1$. To prove that $f^*$ is a cone-net as well consider the functions

$$\begin{aligned} a^*_{ij} = \frac{\lambda _{j + 1} (a_{ij} + 1) - \lambda _j}{\lambda _j}, \qquad b^*_{ij} = \frac{\lambda _{j + 1} (a_{ij} + 1) - \lambda _j}{\lambda _{j + 1} (a_{i - 1, j} + 1) - \lambda _j} - 1. \end{aligned}$$

They satisfy $\delta _i \delta _j f_{ij}^* = a^*_{ij} \delta _i f_{ij}^* + b^*_{ij} \delta _j f_{ij}^*$ and $b^*_{ij} = \frac{a^*_{ij}}{a^*_{i-1,j}} -1$ which implies (by Lemma 19) that $f^*$ is a cone-net.

To prove that the transformed net is parameterized by Eq. (18) we show that the edges of the parameterization are given by Eq. (17). We get:

$$\begin{aligned} \delta _i f_{ij}^*&= f^*_{i + 1, j} - f^*_{ij} \\&= \lambda _j f_{i + 1, j} - \sum _{k = 0}^{j - 1} (\lambda _{k + 1} - \lambda _k) r_k - \lambda _j f_{ij} + \sum _{k = 0}^{j - 1} (\lambda _{k + 1} - \lambda _k) r_k = \lambda _j \delta _i f_{ij} \\ \delta _j f_{ij}^*&= \lambda _{j + 1} f_{i, j + 1} - \sum _{k = 0}^j (\lambda _{k + 1} - \lambda _k) r_k - \lambda _j f_{ij} + \sum _{k = 0}^{j - 1} (\lambda _{k + 1} - \lambda _k) r_k \\&= (\lambda _{j + 1} - \lambda _j) f_{i, j + 1} + \lambda _j \delta _j f_{ij} - (\lambda _{j + 1} - \lambda _j) r_j \\&\overset{(16)}{=} (\lambda _{j + 1} - \lambda _j) f_{i, j + 1} + \lambda _j \delta _j f_{ij} - (\lambda _{j + 1} - \lambda _j) \Big (f_{ij} - \frac{b_{ij} + 1}{a_{ij}} \delta _j f_{ij}\Big ) \\&= \Big ((\lambda _{j + 1} - \lambda _j) \Big (1 + \frac{b_{ij} + 1}{a_{ij}}\Big ) + \lambda _j\Big ) \delta _j f_{ij} = \Big (\lambda _{j + 1} + \frac{\lambda _{j + 1} - \lambda _j}{a_{i - 1, j}}\Big ) \delta _j f_{ij}, \end{aligned}$$

which concludes the proof. $\square$

#### Corollary 21

A CCT with discrete function $\lambda$ scales the polygons $(f_{ij})_i$ by the factor $\lambda _j$.

### Discrete Double Cone-Nets

In analogy to Sect. 2.4 we will investigate discrete nets which are cone-nets in both parameter directions.

#### Definition 11

A discrete conjugate net $f: U \subset \mathbb {Z}^2 \rightarrow \mathbb {R}^3$ is called a *double cone-net* if all horizontal and all vertical strips are contained in discrete cones or cylinders.

**Fig. 6:**
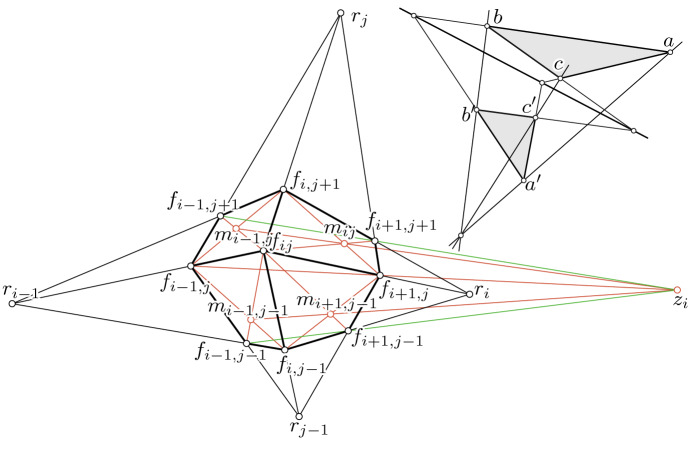
*Left:* Illustration for the proof of Theorem 22: we apply Desargues’ theorem four times. *Top-Right:* Desargue’s theorem

Double cone-nets are related to so called Kœnigs nets. The following characterization of a discrete Kœnigs net can be found in [23]. Since all faces of a discrete conjugate net are planar quadrilaterals, the diagonals of a quadrilateral $q_{ij}$ intersect in a point $m_{ij}$ (see Fig. 6 left).

#### Definition 12

A conjugate net $f: U \rightarrow \mathbb {R}^3$ is called a (discrete) *Kœnigs net* if the three lines

19 $$\begin{aligned} m_{ij} m_{i - 1, j} \quad m_{i + 1, j - 1} m_{i - 1, j - 1} \quad f_{i + 1, j} f_{i - 1, j} \end{aligned}$$

are concurrent.

By swapping *i* and *j* we obtain a condition which is equivalent to the above Kœnigs condition, namely the three straight lines $m_{ij} m_{i + 1, j - 1}$, $m_{i - 1, j} m_{i - 1, j - 1}$, $f_{i, j + 1} f_{i, j - 1}$ meeting in a point. This equivalence is a simple consequence of Desargue’s theorem. *Desargues’ theorem* (see Fig. 6 right) says that two triangles $\Delta (a \mid b \mid c), \Delta (a' \mid b' \mid c')$ are *centrally perspective* (i.e., the three lines $a a', b b', c c'$ are concurrent) if and only if they are *axially perspective* (i.e., the three points $(a b \cap a' b'), (b c \cap b' c'), (c a \cap c' a')$ are collinear).

We will apply Desargues’ theorem a couple of times to show the following two theorems which discretize Theorem 7.

#### Theorem 22

Every double cone-net is a Kœnigs net.

#### Proof

It is sufficient to consider a $2 \times 2$-subpatch of the net (see Fig. 6 left). We have to show that the three lines from Eq. (19) are concurrent. To do so we will apply Desargues’ theorem four times:
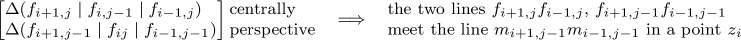

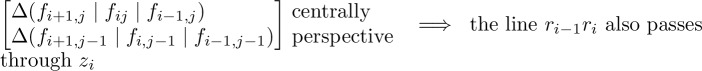

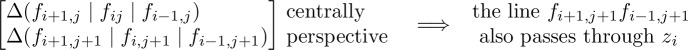

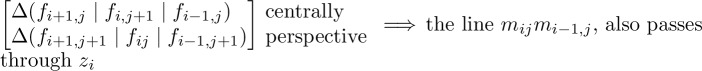


Consequently, the three lines from Eq. (19) are concurrent. $\square$

The converse result holds only in a slightly modified version as we must assume at least one vertical strip to be contained in a cone. There exist Kœnigs nets with horizontal strips being cones but which are not double cone-nets. For such an example see Fig. 5 (right).

#### Theorem 23

A Kœnigs cone-net with horizontal strips being cones is a double cone-net if and only if at least one vertical strip is contained in a cone.

#### Proof

**Fig. 7:**
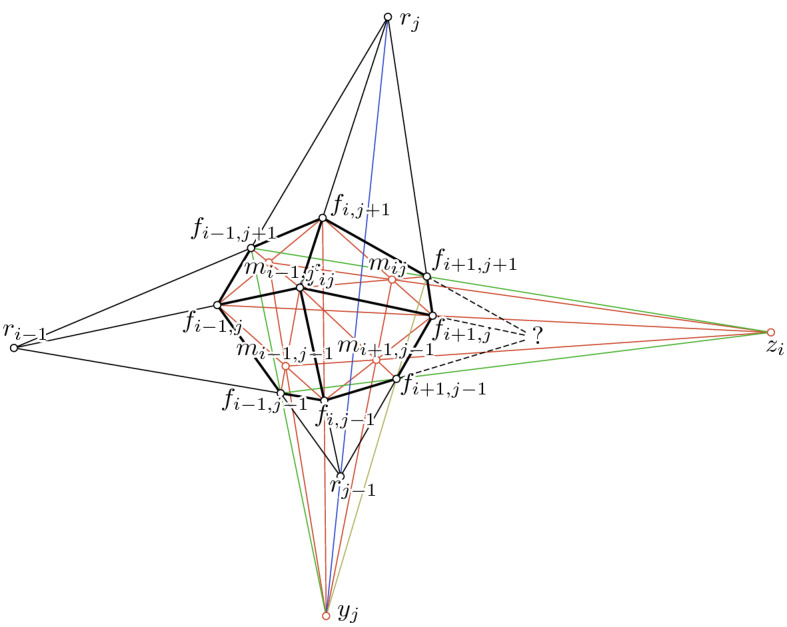
*Left:* Illustration for the proof of Theorem 23: we apply Desargues’ theorem six times

It is sufficient to consider a $2 \times 2$-subpatch of the net (see Fig. 7). We assume the two horizontal strips $B_j$ and $B_{j - 1}$ to be cones with centers $r_j$ and $r_{j - 1}$ and the “left” vertical strip $B^{i - 1}$ to be a cone with center $r_{i - 1}$. Furthermore, we assume the net to be a Kœnigs net.

It is our goal to prove the cone-net-property of the “right” vertical strip $B^i$. For that we apply Desargues’ theorem six times (see Fig. 7 for a reference of notation):
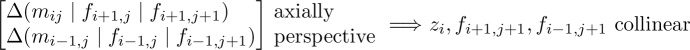

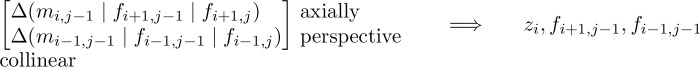

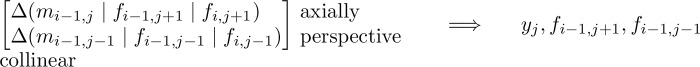

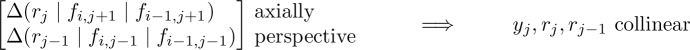

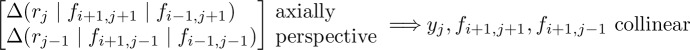

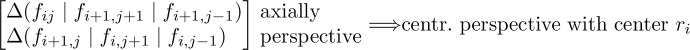


Therefore, the “right” vertical strip $B^i$ is a cone. $\square$

#### Remark 3

An alternative proof of the last theorem in a projective setup using Laplace invariants follows ideas presented in [8]. The *Laplace invariants* are given as cross-ratios of four collinear points (for the definition of the Laplace points $\mathcal {L}^k$ see Remark 2):

$$\begin{aligned} h_{ij} := \textrm{cr}(f_{ij}, \mathcal {L}^1_{ij}, \mathcal {L}^1_{i, j - 1}, f_{i + 1, j}), \qquad k_{ij} := \textrm{cr}(f_{ij}, \mathcal {L}^2_{ij}, \mathcal {L}^2_{i - 1, j}, f_{i, j + 1}). \end{aligned}$$

The net is a cone-net if one of the families of Laplace points degenerates to a polygon, i.e., $\mathcal {L}^1_{ij} = \mathcal {L}^1_{i, j - 1}$ for all *j*, or equivalently, if one of the Laplace invariants has constant value 1, i.e., $h_{ij} = 1$. The cross-ratio is well defined for projective lines and invariant under projective transformations. Therefore, this definition for cone-nets works as well for nets in $\mathbb {P}(\mathbb {R}^3)$. For more information on projective differential geometry and Laplace invariants see [24].

Also Kœnigs nets can be characterized in terms of Laplace invariants. For a discrete Kœnigs net the Laplace invariants satisfy

20 $$\begin{aligned} h_{ij} h_{i - 1, j} = k_{ij} k_{i, j - 1}, \end{aligned}$$

for all *i*, *j* which can be found for instance in [8]. If a Kœnigs net is a cone-net in the horizontal direction, i.e., $h_{ij} = 1$ and has also one vertical cone-strip (e.g., $k_{0, j} = 1$), then Eq. (20) implies that the net is a double cone-net.

#### Remark 4

(Cauchy data for double cone-nets) Any double cone-net over a rectangular domain is uniquely determined by any two cone-strips $B_{i_0}$ and $B_{j_0}$.

Double cone-nets have also been investigated in the framework of so called multi-nets [15].

#### Definition 13

A discrete conjugate net $f: \mathbb {Z}^2 \supset U \rightarrow \mathbb {R}^3$, is called a *multi Q-net*, if for every $i_0 \ne i_1$ and $j_0 \ne j_1$ the quadrilateral $(f_{i_0, j_0}, f_{i_1, j_0}, f_{i_1, j_1}, f_{i_0, j_1})$ is planar. The net *f* is called *multi-circular* if the quadrilaterals $(f_{i_0, j_0}, f_{i_1, j_0}, f_{i_1, j_1}, f_{i_0, j_1})$ have circumcircles (see also Lemma 37).

Bobenko et al.  [15, Th. 2.4] show that a net is a double cone-net if and only if it is a multi Q-net.

### Discrete Principal Cone-Nets

A common discretization of principal nets are nets with $\mathbb {Z}^2$ combinatorics such that every face has a circumcircle [8]. The following definition is not to be confused with so called *conical nets* in [1, 8] which also discretize principal nets.

#### Definition 14

A *discrete principal cone-net* is a discrete cone-net with concyclic faces, i.e., $f_{ij}, f_{i + 1, j}, f_{i + 1, j + 1}, f_{i, j + 1}$ is a concyclic quadrilateral for all *i*, *j* with circumcirle $C_{ij}$, and the edges $f_{ij} \vee f_{i, j + 1}$ pass through a common point $r_j$, for all *i*. If no point $r_j$ is a point at infinity, we call the principle cone-net *proper*.

#### Corollary 24

Discrete conical Combescure transformations map principal cone-nets to principal cone-nets.

#### Proof

Let *f* be a discrete cone-net and $f^*$ a CCT of *f*. Since corresponding edge vectors of *f* and $f^*$ are parallel, the quadrilaterals $q_{ij}$ of *f* have circumcirlces if and only if the quadrilaterals $q_{ij}^*$ of $f^*$ have circumcirlces. $\square$

**Fig. 8:**
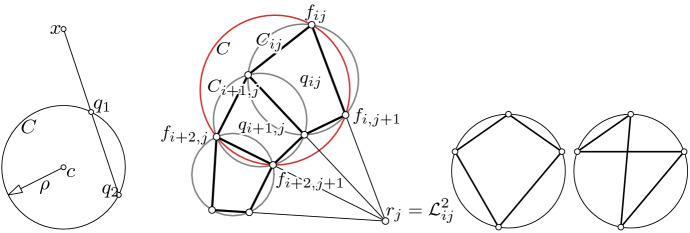
*Left:* Power of a point *x* with respect to a circle *C* with center *c* and radius $\rho$. *Center:* The three radical axes of any pair of three circles (*C* and circumcircles of $q_{ij}$ and $q_{i + 1, j}$) meet in a point. *Right:* An embedded concyclic quadrilateral and a non-embedded concyclic quadrilateral

In our investigation we will take advantage of the concept of the power of a point with respect to a circle (Fig. 8 left). Let *C* be a circle with center *c* and radius $\rho$, and let *x* be a point in the plane carrying that circle. Further, let *l* be a line through *x* intersecting the circle *C* in two points $q_1, q_2$. The *(oriented) power* of the point *x* with respect to the circle *C* is given by (see., e.g., [25])

$$\begin{aligned} p := \langle x - q_1, x - q_2 \rangle . \end{aligned}$$

The power is independent of the choice of the line *l* which implies

$$\begin{aligned} p = (\Vert c - x\Vert + \rho ) (\Vert c - x\Vert - \rho ). \end{aligned}$$

Note that the power is positive if *x* lies outside *C* and negative if *x* lies inside. The *radical axis* of two circles in a plane is the straight line of points with equal power to both circles. If two circles are intersecting, then the radical axis is given by the line through the intersection points. The three radical lines of three circles are either parallel if the three corresponding centers lie on a straight line or otherwise they meet in a point, the so called *radical center* (see, e.g., [25] and Fig. 8 center).

#### Definition 15

For a discrete principle net, we define the *geodesic curvature* $\kappa ^g_{ij}$ of a quadrilateral $q_{ij}$ in a strip $B_j$ via the power of the Laplace point $\mathcal {L}^2_{ij}$ with respect to the circumcircle $C_{ij}$:

$$\begin{aligned} \vert \kappa ^g_{ij}\vert&:= \vert p_{ij}\vert ^{-\frac{1}{2}} := \vert \langle \mathcal {L}^2_{ij} - f_{ij}, \mathcal {L}^2_{ij} - f_{i, j + 1}\rangle \vert ^{-\frac{1}{2}} \\&= \vert \langle \mathcal {L}^2_{ij} - f_{i + 1, j}, \mathcal {L}^2_{ij} - f_{i + 1, j + 1}\rangle \vert ^{-\frac{1}{2}}. \end{aligned}$$

The sign of the geodesic curvature is defined by considering the order of the points. The curvature is *positive* if $f_{i, j + 1}$ is between $f_{ij}$ and $\mathcal {L}^2_{ij}$ and *negative* if $f_{ij}$ is between $f_{i, j + 1}$ and $\mathcal {L}^2_{ij}$.

#### Remark 5

Note, that the powers of both Laplace points $\mathcal {L}^1_{ij}, \mathcal {L}^2_{ij}$ with respect to the circumcircle of the corresponding quadrilateral is positive if and only if the quadrilateral is embedded, cf. Fig. 8 right (i.e., $f_{ij}, f_{i, j + 1}, f_{i + 1, j + 1}, f_{i, j + 1}$ lie on the convex hull of the quadrilateral in cyclic order).

#### Lemma 25

A discrete principle net is a cone-net, if and only if all horizontal or vertical strips have constant geodesic curvature.

#### Proof

Assume the horizontal strips $B_j$ have constant geodesic curvature and consider two neighboring quadrilaterals $q_{ij}, q_{i + 1, j}$ with circumcircles $C_{ij}, C_{i + 1, j}$. Since the quadrilaterals have the same geodesic curvature, we have

$$\begin{aligned}&\langle \mathcal {L}^2_{ij} - f_{i + 1, j}, \mathcal {L}^2_{ij} - f_{i + 1, j + 1} \rangle ^{-\frac{1}{2}} = \langle \mathcal {L}^2_{ij} - f_{ij}, \mathcal {L}^2_{ij} - f_{i, j + 1} \rangle ^{-\frac{1}{2}} \\&\quad = \kappa ^g_{ij} = \kappa ^g_{i + 1, j} = \langle \mathcal {L}^2_{i + 1, j} - f_{i + 1, j}, \mathcal {L}^2_{i + 1, j} - f_{i + 1, j + 1} \rangle ^{-\frac{1}{2}}. \end{aligned}$$

Therefore, the Laplace points agree, i.e., $\mathcal {L}^2_{ij} = \mathcal {L}^2_{i + 1, j}$. Induction on *i* implies that the strip $B_j$ is a cone.

Now, we assume that the strips $B_j$ are cones. The cone tip $r_j = \mathcal {L}^2_{ij}$ has the same power with respect to all circumcircles $C_{ij}$ of the strip $B_j$:

$$\begin{aligned} p_j = \langle r_j - f_{ij}, r_j - f_{i, j + 1}\rangle = \langle r_j - f_{i + 1, j}, r_j - f_{i + 1, j + 1}\rangle . \end{aligned}$$

Therefore, the geodesic curvature $\kappa ^g_{ij} = p_j^{-\frac{1}{2}}$ is constant for all quadrilaterals of the strip $B_j$. $\square$

Note that for a principle cone-net, the cone tip $r_j$ is the radical center for any three circumcircles of the strip $B_j$ which leads to the following definition.

#### Definition 16

We call the sphere with center $r_j$ and radius $1/\kappa ^g_{ij}$ *geodesic curvature sphere* and denote it by $S^g_j$.

#### Lemma 26

Let *f* be a discrete principle cone-net and $f^*$ its CCT with respect to $\lambda$, then the geodesic curvature of the cone-strips $B_j$ change according to

$$\begin{aligned} \vert \kappa ^{g\, *}_{ij}\vert = \frac{\vert \kappa ^g_{ij}\vert }{\vert \lambda _j \lambda _{j + 1}\vert ^{1/2}}. \end{aligned}$$

#### Proof

The CCT scales the boundary polygons of the cone-strip $B_j$ by $\lambda _j$ resp. $\lambda _{j + 1}$, (see Corollary 21). Since corresponding edge vectors of *f* and $f^*$ are parallel, the distance between the polygons and the cone tip $r_j$ get scaled by the same factor. Therefore

$$\begin{aligned}&\Vert r_j^*- f_{ij}^*\Vert = \vert \lambda _j\vert \, \Vert r_j-f_{ij}\Vert \\ \Rightarrow \qquad&\vert p_j^*\vert = \Vert r_j^*- f_{ij}^*\Vert \, \Vert r_j^* - f_{i,j+1}^*\Vert = \vert \lambda _j \lambda _{j + 1}\vert \, \Vert r_j - f_{ij}\Vert \, \Vert r_j - f_{i, j + 1}\Vert \\ \Rightarrow \qquad&\vert \kappa ^{g\, *}_{ij}\vert = \frac{1}{\vert \lambda _j \lambda _{j + 1}\vert ^{1/2}} \vert \kappa ^g_{ij}\vert . \end{aligned}$$

$$\square $$

#### Lemma 27

Let *f* be a principle cone-net and let *j* be fixed. Then the inversion at the geodesic curvature spheres $S^g_j$ preserves the circumcircles $\{C_{ij}\}_i$ and maps the boundary polygons $(f_{ij})_i$ and $(f_{i, j + 1})_i$ of the strip $B_j$ onto each other. In particular, $S^g_j$ intersects the circumcircles $\{C_{ij}\}_i$ and the edges $\{f_{ij} \vee f_{i, j + 1}\}_i$ orthogonally.

#### Proof

Since all faces of the strip are concyclic, the oriented power of $r_j$ with respect to the two corresponding vertices on these edges is constant

$$\begin{aligned} \langle f_{ij} - r_j, f_{i, j + 1} - r_j\rangle = p_j = \text {const} \quad \text {for all}\ i, \end{aligned}$$

and therefore

$$\begin{aligned} \langle f_{ij} - r_j, f_{i, j + 1} - r_j\rangle (f_{i, j + 1} - r_j) = p_j (f_{i, j + 1} - r_j). \end{aligned}$$

From the identity $\frac{f_{i, j + 1} - r_j}{\Vert f_{i, j + 1} - r_j\Vert } = \frac{f_{ij} - r_j}{\Vert f_{ij} - r_j\Vert } \frac{\langle f_{i, j + 1} - r_j, f_{ij} - r_j\rangle }{\Vert f_{i, j + 1} - r_j\Vert \Vert f_{ij} - r_j\Vert }$ we conclude

21 $$\begin{aligned} f_{i, j + 1} = p_j \frac{f_{ij} - r_j}{\Vert f_{ij} - r_j\Vert ^2} + r_j. \end{aligned}$$

This equation represents an inversion in the geodesic curvature spheres $S^g_j$ with center $r_j$ and radius $\sqrt{p_j}$.

Since the lines $\{L_{ij}\}_i$ meet in the center of the sphere $S^g_j$, they intersect $S^g_j$ orthogonally and the lines are mapped to themselves under the inversion above. Therefore, the quadrilaterals $\{q_{ij}\}_i$ and their circumcircles are preserved by the inversion. This is the case if and only if the circumcircles $\{C_{ij}\}_i$ intersect the sphere $S^g_j$ orthogonally. $\square$

Since Möbius transformations map spheres to spheres and preserve angles, we obtain the following corollary.

#### Corollary 28

A Möbius transformation applied to the vertices of a discrete principal cone-net is a discrete principal cone-net.

Note that the new cone tips are in general not obtained by the Möbius transformation applied to the old cone tips. They are the centers of the new geodesic curvature spheres.

#### Remark 6

(Cauchy data for principle cone-nets) Suppose we are given a principle cone-net with horizontal cone-strips $B_j$ and constant geodesic curvature spheres $S^g_j$. Then the coordinate polygons $(f_{ij})_j$ are orthogonal trajectories of the geodesic curvature spheres $S^g_j$. In particular, the surface *f* is uniquely determined by an initial polygon $(f_{i, 0})_i$ and the family of geodesic curvature spheres $S^g_j$.

#### Lemma 29

Any four points $f_{ij}, f_{i, j + 1}, f_{i + k, j}, f_{i + k, j + 1}$ of a cone-strip of a principle cone-net are concyclic (see Fig. 8 center). Further, the cone tip $r_j$ has the same power with respect to this circle as to any circumcircle of the strip.

#### Proof

Consider the circle *C* through the three points $f_{ij}, f_{i, j + 1}, f_{i + k, j}$. The power of the cone tip $r_j$ with respect to the circle *C* is the same with respect to any circumcircle of the strip. Therefore, the line through $r_j$ and $f_{i + k, j}$ intersects *C* in the point $f_{i + k, j + 1}$. $\square$

#### Discrete Tractrix Surfaces I

There are several discretizations of a tractrix curve (see, e.g., [26] where discretizations of the hyperbolic cosine are constructed from several discretizations of a tractrix). The following is based on a Darboux transformation for discrete curves [27].

##### Definition 17

**Fig. 9:**
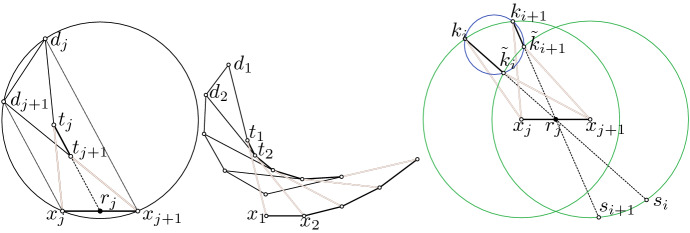
*Left:* Discrete tractrix construction (cf. Definition 17) with base curve $x_j$ and tractrix $t_j$. *Center:* The base curve $x_i$, its Darboux transform $d_i$, and the tractrix $t_i$. *Right:* Illustration for the proof of Lemma 30: If two points $k_i, k_{i + 1}$ are at the same distance to $x_j$, then the next vertices ${{\tilde{k}}}_i, {{\tilde{k}}}_{i + 1}$ in the respective tractrices are concyclic with $k_i, k_{i + 1}$

Let $x_j, d_j \in \mathbb {R}^3$ be two polygons such that $\Vert \delta _j x_j\Vert = \Vert \delta _j d_j\Vert$, $\Vert x_j - d_j\Vert = \text {const}$ and the quadrilateral $x_j, x_{j + 1}, d_{j + 1}, d_j$ lies in a plane but does not form a parallelogram. Then the polygons $x_j$ and $d_j$ are *Darboux transforms* of each other and $t_j:= \frac{1}{2} (x_j + d_j)$ is called *discrete tractrix* with *base polygon* $x_j$ or $d_j$ (see Fig. 9 left and center).

We will define discrete tractrix surfaces in close analogy to Sect. 2.5.1.

##### Definition 18

**Fig. 10:**
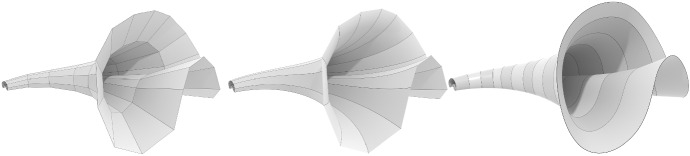
Three discretized tractrix surfaces. A (fully) discrete tractrix surface with planar quadrilateral faces (*left*), two semi-discrete tractrix surfaces of the types $f: \mathbb {Z}\times \mathbb {R}\rightarrow \mathbb {R}^3$ and $f: \mathbb {R}\times \mathbb {Z}\rightarrow \mathbb {R}^3$, i.e., smooth and discrete directions are reversed (*center and right*)

Let $x_j, k_i \in \mathbb {R}^3$ be two polygons and let us consider through each vertex of $k_i$ the discrete tractrix with base curve $x_j$. Then the discrete net formed by these tractrices is called a *generalized discrete tractrix surface*. If the vertices of the initial polygon $k_i$ lie on a sphere around the first point of the base curve, we call the net *discrete tractrix surface* (see Fig. 10).

##### Lemma 30

The generalized discrete tractrix surface is a discrete conjugate net (i.e., a net with planar faces).

##### Proof

Since $t_j$ is the midpoint of $x_j d_j$ and $t_{j + 1}$ is the midpoint of $x_{j + 1} d_{j + 1}$, the connecting line $t_j t_{j + 1}$ passes through $r_j$ which is the midpoint of $x_j x_{j + 1}$ (Fig. 9 left). This property does not depend on the initial vertex position of $t_j$. Therefore, the quadrilateral generated by this tractrix construction through $k_i$ and $k_{i + 1}$ generates a quadrilateral $k_i, k_{i + 1}, {{\tilde{k}}}_{i + 1}, {{\tilde{k}}}_i$ with edges $k_i {{\tilde{k}}}_i$ and $k_i {{\tilde{k}}}_i$ passing through a common point $r_j$ (see Fig. 9 right). This quadrilateral must therefore be planar. Consequently, all quadrilaterals of the net are planar. $\square$

All (generalized) tractrix surfaces are cone-nets with discrete cones along horizontal strips and with cone tips $r_j$.

##### Lemma 31

Any tractrix surface is a discrete principal net with concyclic faces, i.e., if the initial polygon $k_i$ in the construction lies on a sphere with center $x_j$ then the quadrilaterals of the net have circumcircles.

##### Proof

This follows from elementary geometric properties of the power of a point with respect to a circle. We have (see Fig. 9 right)

$$\begin{aligned} \langle r_j - k_i, r_j - {{\tilde{k}}}_i\rangle = -\langle r_j - s_i, r_j - {{\tilde{k}}}_i\rangle =: p_j \end{aligned}$$

and

$$\begin{aligned} \langle r_j - k_{i + 1}, r_j - {{\tilde{k}}}_{i + 1}\rangle = -\langle r_j - s_{i + 1}, r_j - {{\tilde{k}}}_{i + 1}\rangle = p_j, \end{aligned}$$

which both equal $p_j$ since the power $p_j$ of the point $r_j$ with respect to the circumcircle of $s_i, s_{i + 1}, {{\tilde{k}}}_i, {{\tilde{k}}}_{i + 1}$ does not depend on the secant. Therefore, we obtain

$$\begin{aligned} \langle r_j - k_i, r_j - {{\tilde{k}}}_i\rangle = \langle r_j - k_{i + 1}, r_j - {{\tilde{k}}}_{i + 1}\rangle , \end{aligned}$$

which is only possible if the four points $k_i, k_{i + 1}, {{\tilde{k}}}_{i + 1}, {{\tilde{k}}}_i$ lie on a circle. $\square$

In analogy to Corollary 12 we can show the following two theorems.

##### Theorem 32

Any CCT of a discrete tractrix surface is a proper discrete principal cone-net.

##### Proof

Let *f* be a discrete tractrix surface. Theorem 20 implies that any CCT of a discrete tractrix surface is a discrete cone-net. The transformed net is also a principal net since all edge-wise parallel quadrilaterals of a concyclic quadrilateral are concyclic. $\square$

##### Theorem 33

Any proper discrete principal cone-net with spherical parameter polygons $(f_{ij})_i$ is a CCT of a discrete tractrix surface.

##### Proof

Let *f* be a discrete principal cone-net with spherical parameter polygons $(f_{ij})_i$. Furthermore, let $S_j^f$ be the sphere with radius $R_j$ containing the polygon $(f_{ij})_i$. The CCT with $\lambda _j = \frac{1}{R_j}$ transforms *f* into a net $h:= \mathop {\mathcal {C}}\nolimits _{\frac{1}{R_j}}(f)$ where every polygon $(h_{ij})_i$ is obtained by a scaling of $(f_{ij})_i$ with factor $\frac{1}{R_j}$ (up to translation). The corresponding spheres $S_j^h$ which contain $(h_{ij})_i$ are obtained from $S_j^h$ by scaling with the same factor $\frac{1}{R_j}$ and are therefore unit spheres. Let $r^h_j$ denote the cone tips of *h*. The inversion in the geodesic curvature sphere of *h* centered at cone tips $r^h_j$ maps $S^h_j$ to $S^h_{j + 1}$. Since the two spheres are unit spheres, $r^h_j$ must be the midpoint of the two centers of the spheres. The construction from the spherical polygon $(h_{ij})_i$ to the polygon $(h_{i, j + 1})$ corresponds to the tractrix construction (see Fig. 9 right). Consequently, *h* is a tractrix surface. And $f = \mathop {\mathcal {C}}\nolimits _{R_j}(h)$ which concludes the proof. $\square$

#### Discrete Tractrix Surfaces II

We obtain another discretization of a tractrix surface by discretizing the characterizing property of Lemma 10. A tractrix surface is parametrized by a family of orthogonal trajectories of a one-parameter family of spheres with constant radii. Lemma 27 readily provides us with the discrete trajectory construction.

##### Definition 19

Let $f_{i, 0}$ be a polygon and $S^g_j$ be a sequence of geodesic curvature spheres of constant radii. Then the successive inversion of the initial polygon in the geodesic curvature spheres generates a discrete *tractrix-II* surface.

Two endpoints $f_{i, 0}, f_{i + 1, 0}$ of an edge together with their reflections $f_{i, 1}, f_{i + 1, 1}$ in the first geodesic curvature sphere generate a concyclic quadrilateral. The straight lines $f_{i, 0} \vee f_{i, 1}$ and $f_{i + 1, 0} \vee f_{i + 1, 1}$ pass through the center of the geodesic curvature sphere. Therefore, the tractrix-II surface *f* is a principal cone-net.

Consequently, all results obtained so far for discrete principal cone-nets hold for this type of tractrix-II surface. The main difference to the previous definition of tractrix surfaces is that the discrete parameter curves $(f_{ij})_i$ do not necessarily lie on a sphere.

We therefore obtain a theorem in analogy to Theorem 33 but without the requirement of spherical parameter polygons.

##### Theorem 34

Any proper discrete principal cone-net is a CCT of a discrete tractrix-II surface.

##### Proof

The radius of the geodesic curvature sphere of a proper discrete principal cone-net is $1/\vert \kappa _{ij}^g\vert$. Therefore, Lemma 26 yields the radii of the transformed geodesic curvature spheres after a CCT with function $\lambda$.

Consequently, $\lambda$ can be chosen in such a way that $\vert \kappa _{ij}^{g\, *}\vert$ is constant for all *j* which implies that $f^*$ is a tractrix-II surface. $\square$

**Fig. 11:**
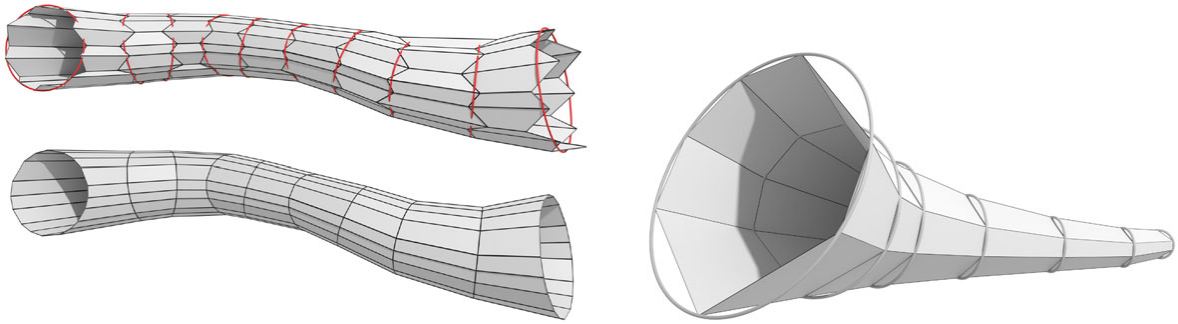
*Top-left:* A discrete canal surfaces as in Definition 20. The “zig-zaggy” parameterlines are spherical and have constant curvature circles (red). *Bottom-left:* The vertices that define the curvature circles of the discrete canal surface (*top-left*) constitute a discrete canal surface by themselves but with concyclic parameter polygons. *Right:* A discrete canal surface with concyclic parameter polygons as in [14]

#### Discrete Canal Surfaces

Stripmodels from annulus-shaped strips of surfaces of revolution have been studied, e.g., in [5, 6]. These are (semi-)discretizations of a particular class of canal surfaces. Discrete canal surfaces have recently been revisited in [14]. In the present subsection we will give a novel and more flexible definition of canal surfaces that includes the discrete canal surfaces from [14]. Our definition is based on the notion of a Möbius invariant definition of a curvature circle for discrete curves [28].

The following notions are explained in more detail in [28]. Let us identify $\mathbb {R}^3$ with the imaginary part $\mathop {\text {Im}}\mathbb {H}$ of the quaternions $\mathbb {H}$. Then four points *a*, *b*, *c*, *d* have the cross-ratio $\mathop {\text {cr}}(a, b, c, d):= (a - b) (b - c)^{-1} (c - d) (d - a)^{-1}$. A new point is computed by

$$\begin{aligned}&p(a, b, c, d) \\ {}&\quad := \big ((b - a) (c - a)^{-1} \sqrt{\mathop {\text {cr}}(c, a, b, d)} + 1 \big )^{-1} \big ((b - a) (c - a)^{-1} \sqrt{\mathop {\text {cr}}(c, a, b, d)} c + b \big ). \end{aligned}$$

It turns out [28, Cor. 4] that *p*(*a*, *b*, *c*, *d*) always lies on the circumsphere of *a*, *b*, *c*, *d* and that the four points *p*(*a*, *b*, *c*, *d*), *p*(*b*, *c*, *d*, *a*), *p*(*c*, *d*, *a*, *b*), *p*(*d*, *a*, *b*, *c*) always lie on a circle [28, Th. 1]. If *a*, *b*, *c*, *d* are four successive points of a discrete curve then this circle can be interpreted as a *curvature circle* for the discrete curve at edge *bc* [28, Th. 4]. If *a*, *b*, *c*, *d* lie on a circle then this circle is identical to the curvature circle [28, Cor. 1].

Since the curvature lines of a smooth canal surface are circles and since a curve with a constant curvature circle must be a circle, we impose in the following definition on our discrete parameter curves in circular direction to have a constant curvature circle.

##### Definition 20

A *discrete canal* surface is a principal cone-net *f* with spherical parameter curves $(f_{ij})_i$ with constant curvature circles. We call the reciprocals of the radii of the spheres *discrete principal curvatures (in circle direction)* $\kappa _1(j)$.

Note that discrete parameter curves in circular directions of canal surfaces in our definition are not necessarily concyclic even though the curvature circle is constant along the curve. However, since the curvature circle of a concyclic polygon equals the circumcircle, the canal surfaces from [14] constitute a subclass of ours as there concyclic parameter curves are required (see Fig.  11 for illustrations of discrete canal surfaces).

In analogy to Theorem 13 discrete canal surfaces are characterized by their Gauss image. The discrete Gauss image of a net *f* is (in our setting) a net which is edge-wise parallel to *f* with vertices on a sphere (cf. [8]).

##### Theorem 35

A discrete principal cone-net is a canal surface if and only if its Gauss image is a canal surface.

##### Proof

Lemma 27 implies that the polygons $(f_{ij})_i$ and $(f_{i, j + 1})_i$ are mapped onto each other by inversion in the geodesic curvature sphere $S_j^g$ with center $r_j$. Therefore, the sphere containing $(f_{i, j + 1})_i$ can be mapped to the sphere $\mathcal {S}$ containing $(f_{ij})_i$ by a homothety. Applying this homothety to the entire net yields a net with two co-spherical parameter polygons on $\mathcal {S}$. In this way we generated the Gauss image of the first strip $B_j$ since all edges are parallel to the corresponding edges of the original net.

We can continue by mapping the next parameter curve $(f_{i, j + 2})_i$ to $\mathcal {S}$ with another homothety. In this way by continuing we obtain the Gauss image.

Since corresponding parameter curves of *f* and its Gauss image only differ by a homothety (which is a Möbius transformation) either both have a constant curvature circle or none. $\square$

##### Definition 21

A *(vertex) offset* $f^d$ of a discrete principal net *f* has the same combinatorics as *f* such that corresponding edges are parallel and the distance between corresponding vertices is constant.

For nets over a simply connected domain the existence of a vertex offset net is equivalent to the existence of an edgewise parallel net $(f^d - f)/d$ inscribed into the unit sphere [29] which is the discrete Gauss image. Note that the set of edgewise parallel nets is a vector space with vertex-wise addition and scalar multiplication.

##### Theorem 36

Let *f* be a discrete canal surface and $\kappa _1(j)$ the discrete principle curvature of the discrete circular parameter curves. The offset $f^d$ is up to translation a CCT of *f* for $\lambda _j = 1 + d\,\kappa _1(j)$.

##### Proof

By the proof of Theorem 35 the Gauss image *n* exists and corresponding *i*-parameter curves are related by a homothety. The scaling factor from $(f_{ij})_i$ to its Gauss image $(n_{ij})_i$ is $\kappa _1(j)$. Consequently,

$$\begin{aligned} f^d = f + d\, n \end{aligned}$$

implies

$$\begin{aligned} (f_{ij})_i^d = (f_{ij})_i + d\, (n_{ij})_i = (f_{ij})_i + d\, \kappa _1(j) (f_{ij})_i = (1 + d\, \kappa _1(j)) (f_{ij})_i, \end{aligned}$$

which concludes the proof. $\square$

##### Lemma 37

Any double principle cone-net $f:\mathbb {Z}^2 \rightarrow \mathbb {R}^3$ is a multi-circular net (cf. Definition 13).

##### Proof

Bobenko et al.  [15, Th. 2.4] show that any double cone-net is a multi-cone-net.

Moreover, Lemma 29 implies that any strip $B_j$ is a multi-circular strip in itself.

Consequently, the strip $(f_{i_0, j}, f_{i_1, j}, f_{i_1, j + 1}, f_{i_0, j + 1})_j$ is a circular cone-strip and by Lemma 29 a multi-circular strip. Hence, the quadrilateral $(f_{i_0, j_0}, f_{i_1, j_0}, f_{i_1, j_1}, f_{i_0, j_1})$ is concyclic and therefore the net is multi-circular. $\square$

In analogy to Theorem 16 we obtain:

##### Theorem 38

Every discrete double principal cone-net is Möbius equivalent to a surface of revolution, cone or cylinder.

##### Proof

Lemma 37 implies that the net is multi-circular. Bobenko et al. [15, Th. 7.7] show that multi-circular nets are Möbius equivalent to surfaces of revolution, cones or cylinders. $\square$

## Figures and Tables

**Fig. 12:**
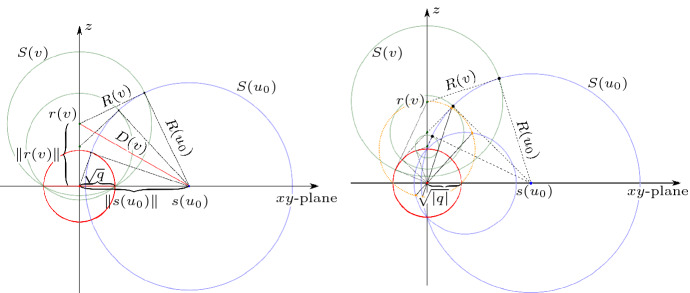
Two families of orthogonally intersecting spheres. The centers of one of the families lie on the *z*-axis and the centers of the other family in the *xy*-plane. *Left:* The sphere with radius $\sqrt{q}$ centered at the origin gets intersected orthogonal by all spheres *S*(*u*) while all the spheres *S*(*v*) intersect that sphere in the same circle lying in the *xy*-plane. *Right:* The sphere with radius $\sqrt{\vert q\vert }$ centered at the origin, gets intersected orthogonally by all spheres *S*(*v*) while all the spheres *S*(*u*) contain the north and south pole of the that sphere

## Appendix A Proof of Theorem 16

### Proof of Theorem 16

We prove the statement by analyzing three cases. Before we start with that we compute the derivatives of the curves traced out by the cone tips.

Let $f: U \rightarrow \mathbb {R}^3$ be a double principal cone-net. Theorem 7 implies that *f* is a Kœnigs net and orthogonal Kœnigs nets are isothermic. After a possible reparameterization ($u \mapsto {\tilde{u}}(u), v \mapsto {\tilde{v}}(v)$), we can assume that the parameterization is conformal, i.e., $\alpha = \Vert f_u\Vert = \Vert f_v\Vert = \beta$. Then we have

A1 $$\begin{aligned} \frac{\beta _v}{\beta } = \frac{\alpha _v}{\alpha } \overset{(6)}{=} \frac{-\beta \kappa }{\alpha } = -\kappa , \quad \quad \frac{\alpha _u}{\alpha } = \frac{\beta _u}{\beta } \overset{(7)}{=} \frac{\alpha \eta }{\beta } = \eta . \end{aligned}$$

For coordinate curves with non-vanishing geodesic curvature we denote the tips of the cones along the *u*-parameter curves by *r*(*v*) and the tips of the cones tangent to the *v*-parameter curves by *s*(*u*). Using Eq. (2) and the frame Eqs. (5) we compute the curve of cone tips

$$\begin{aligned} r(v) \overset{(12)}{=} f(u, v) + \frac{\alpha }{\kappa } Y(u, v) \quad \text {and analogously}\quad s(u) = f(u, v) - \frac{\alpha }{\eta } X(u, v), \end{aligned}$$

and their derivatives:

A2 $$\begin{aligned} r_v(v)&\overset{(5)}{=} -\frac{\eta \alpha }{\kappa } X + \big (\alpha + \Big (\frac{\alpha }{\kappa }\Big )_v \big ) Y + \frac{d \alpha }{\kappa } N \overset{(A1)}{=} \frac{\alpha }{\kappa } \big ( -\eta X -\frac{\kappa _v}{\kappa } Y + d N\big ) \nonumber \\ s_u(u)&\overset{(5)}{=} \big (\alpha - \Big (\frac{\alpha }{\eta }\Big )_u \big ) X - \frac{\kappa \alpha }{\eta } Y - \frac{c \alpha }{\eta } N \overset{(A1)}{=} \frac{\alpha }{\eta }\Big ( \frac{\eta _u}{\eta } X -\kappa Y + - c N \Big ). \end{aligned}$$

After this preparatory work we start to analyze three cases depending on the values of the geodesic curvature of the parameter lines. First, note that the (generalized) spheres of the family *S*(*u*) with centers *s*(*u*) and radii $\vert k_g^v\vert ^{-1}$ intersect the (generalized) spheres of the family *S*(*v*) with centers *r*(*v*) and radii $\vert k_g^u\vert ^{-1}$ orthogonally since $f(u, v) - s(u) \perp f(u, v) - r(v)$.

*—Case 1:* All parameter curves are geodesics. Consequently, $\eta = \kappa = 0$. From the Gauss Eq. (8), we obtain $0= \kappa _v -\eta _u = cd$. If $c = 0$, we further have $X_v=X_u=Y_u=0$ (cf. (5)), hence the *u*-curves are straight lines and the surface is a cylinder over the *v*-curves. The case $d = 0$ works analogously.

*—Case 2:* The *u*-curves are geodesics and there exist *v*-curves that are not geodesics. Consequently, $\kappa = 0$. Due to Lemma 8 and its proof the geodesics are contained in the planes $S(v) = \{x \in \mathbb {R}^3 \mid \langle x - f(u, v), Y(v) \rangle \}$ which intersect the spheres *S*(*u*) orthogonally. Therefore, *S*(*v*) contains the centers of *S*(*u*).

All planes *S*(*v*) are orthogonal to any sphere *S*(*u*) hence they all pass through their centers *s*(*u*). Let us choose an arbitrary sphere $S(u_0)$. Consequently, all planes *S*(*v*) pass through $s(u_0)$. Now, either all these planes only share one point, $s(u_0)$, or they share a straight line passing through $s(u_0)$.

In the first case the spheres *S*(*u*) are concentric which implies $s_u(u) = 0$ and Eq. (A2) yields $\eta _u = c = 0$. This implies $X_u = 0$ (cf. (5)) and the *u*-curves are straight lines that meet in the common center of the spheres. The surface is therefore a cone.

In the second case the centers of *S*(*u*) lie on a straight line *s*(*u*). From $\kappa = 0$ we obtain that *Y* is orthogonal to $s_u(u)$ (see Eq. (A2)). This implies that the *v*-curves lie in planes orthogonal to $s_u(u)$ and the surface is a surface of revolution.

*—Case 3:* Both families of parameter curves contain curves with non-vanishing geodesic curvature. The Gauss Equation (8) implies that the tangent vectors of the curves of cone tips are orthogonal at every point

$$\begin{aligned} \langle r_v(v), s_u(u)\rangle = \frac{\alpha }{\kappa } \frac{\alpha }{\eta } (-\eta _u + \kappa _v - c d) = 0. \end{aligned}$$

Note that neither $r_v(v)$ nor $s_u(u)$ can be zero because if one family of spheres is concentric, then the other family has to consist of planes containing the center. This would contradict the assumption, that both families of parameter curves contain curves with non-vanishing geodesic curvature. Hence, one of the curves is a straight line and the other one lies in a plane orthogonal to this line. W.l.o.g., we assume that *r*(*v*) is contained in the *z*-axis and that *s*(*u*) is contained in the *xy*-plane. We choose a sphere $S(u_0)$ with radius $R(u_0) = (\kappa _g^{u_0})^{-1}$ and center $s(u_0)$ in the *xy*-plane. For every sphere *S*(*v*) we denote the distance of its center *r*(*v*) to the center $s(u_0)$ by *D*(*v*) (see Fig. 12). Pythagoras’ theorem implies

$$\begin{aligned}&\Vert s(u_0)\Vert ^2 + \Vert r(v)\Vert ^2 = D(v)^2 = R^2(v) + R(u_0)^2 \\ \Leftrightarrow \qquad&R^2(v) - \Vert r(v)\Vert ^2 = \Vert s(u_0)\Vert ^2 - R(u_0)^2 =: q , \end{aligned}$$

for some $q \in \mathbb {R}$. If $q > 0$ (Fig. 12 left), the last equation implies that all spheres *S*(*v*) intersect the *xy*-plane in the same circle $\{(x, y, 0) \in \mathbb {R}^3 \mid x^2 + y^2 = q\}$. After applying a Möbius transformation that maps this circle to a straight line the spheres *S*(*v*) become planes that intersect in that line and we are in the same situation as considered in Case 2. If $q < 0$ (Fig. 12 right), all the spheres *S*(*v*) intersect the sphere $K:= \{(x, y, z) \in \mathbb {R}^3 \mid x^2 + y^2 + z^2 = -q\}$ orthogonally and the spheres *S*(*u*) intersect the *z*-axis in the points $(0, 0, \pm q)$. The inversion in the sphere with center $(0, 0, -q)$ and radius $\sqrt{-2 q}$, maps the sphere *K* to the *xy*-plane and the *z*-axis to itself. Since the spheres *S*(*v*) intersect *K* and the *z*-axis orthogonally, their images under the inversion are spheres centered at the origin. The spheres *S*(*u*) get mapped to planes containing the origin because they contain the points $(0, 0, \pm q)$. Hence, we are again in the same situation as discussed in Case 2. If $q = 0$, all spheres contain the origin. An inversion at any sphere with the origin as center maps all the spheres to planes. This situation was considered in Case 1. $\square$
